# Unveiling the multifaceted antiproliferative efficacy of *Cichorium endivia* root extract by dual modulation of apoptotic and inflammatory genes, inducing cell cycle arrest, and targeting COX-2†

**DOI:** 10.1039/d4ra02131b

**Published:** 2024-06-17

**Authors:** Abdullah R. Alzahrani, Nora Hosny, Doaa I. Mohamed, Hebatallah H. Abo Nahas, Abdulaziz Albogami, Tahani Mohamed Ibrahim Al-Hazani, Ibrahim Abdel Aziz Ibrahim, Alaa Hisham Falemban, Ghazi A. Bamagous, Essa M. Saied

**Affiliations:** a Department of Pharmacology and Toxicology, Faculty of Medicine, Umm Al-Qura University Makkah Saudi Arabia aralzahrani@uqu.edu.sa iamustafa@uqu.edu.sa ahfalemban@uqu.edu.sa gabamagous@uqu.edu.sa; b Medical Biochemistry and Molecular Biology Department, Faculty of Medicine, Suez Canal University Ismailia 41522 Egypt Ahmedn@umn.edu; c Center of Excellence in Molecular and Cellular Medicine, Faculty of Medicine, Suez Canal University Ismailia Egypt; d Department of Clinical Pharmacology and Therapeutics, Faculty of Medicine, Ain Shams University Cairo 11566 Egypt doaapharma@med.asu.edu.eg; e Zoology Department, Faculty of Science, Port Said University Port Said 42526 Egypt hebatallah_hassan@science.suez.edu.eg; f Biology Department, Faculty of Science, Al-Baha University Al Aqiq Saudi Arabia aalbogami@bu.edu.sa; g Biology Department, College of Science and Humanities, Prince Sattam bin Abdulaziz University P. O. Box: 83 Al-Kharj 11940 Saudi Arabia t.alhazani@psau.edu.sa; h Chemistry Department, Faculty of Science, Suez Canal University 41522 Ismailia Egypt; i Institute for Chemistry, Humboldt Universität zu Berlin 12489 Berlin Germany saiedess@hu-berlin.de

## Abstract

Chicory (*Cichorium endivia* L. *divaricatum*) is a renowned medicinal plant traditionally used for various ailments, yet the pharmacological potential of its roots, particularly in terms of antitumor activity, remains elusive. In the present study, we explore, for the first time, the metabolomic profile of ethanolic extract from *Cichorium endivia* roots (CIR) and further unveil its antiproliferative potential. The untargeted phytochemical analysis UPLC/T-TOF-MS/MS identified 131 metabolites in the CIR extract, covering acids, amino acids, flavonoids, alkaloids, nucleotides, and carbohydrates. The antiproliferative activity of the CIR extract was tested in 14 cancer cell lines, revealing significant cytotoxicity (IC_50_: 2.85–29.15 μg mL^−1^) and a high selectivity index. Among the cells examined, the CIR extract recorded the most potent antiproliferative activity and selectivity toward HepG2 and Panc-1 cells, with an IC_50_ of 2.85 μg mL^−1^ and 3.86 μg mL^−1^, respectively, and SI > 10. Insights into the mode of action of the antiproliferative activity revealed that CIR extract induces cell arrest in the S phase while diminishing cell distribution in the G0/G1 and G2/M phases in HepG-2 and Panc-1 cells. Flow cytometric and RT-PCR analysis revealed that the CIR extract significantly triggers apoptosis and modulates the expression of pro-apoptotic and anti-apoptotic genes. Furthermore, the CIR extract exhibited a pronounced anti-inflammatory activity, as evidenced by down-regulating key cytokines in LPS-induced RAW 264.7 cells and selectively inhibiting the COX-2 enzyme. Finally, the CIR extract showed a robust total antioxidant capacity, together with potent free radicals and metal scavenging properties, highlighting its role in alleviating oxidative stress. Taken together, this study highlights the multifaceted therapeutic potential of CIR extract as a natural-based antitumor supplement.

## Abstract 2

Delving into the therapeutical potential of *Cichorium endivia* root extract as a natural-based antitumor supplement.

## Graphical abstract

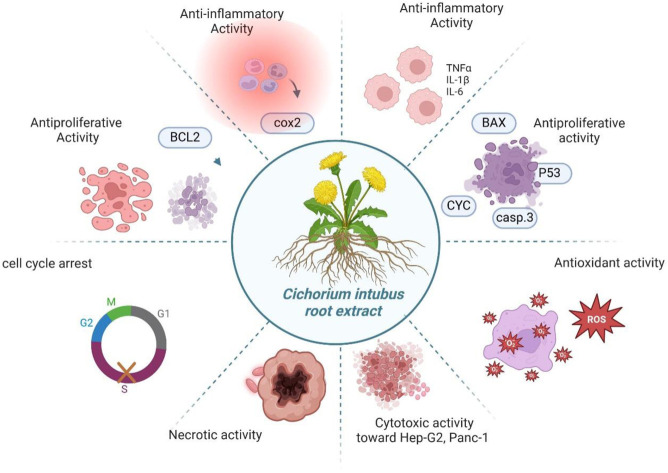

## 1 Introduction

Cancer is one of the leading causes of premature death worldwide and its burden is expected to double by 2070 compared to 2020, with an estimated 34 million new cases in 2070.^1^ Hepatocellular carcinoma (HCC), the primary type of liver cancer, ranks globally as the fourth leading cause of cancer-related death. The burden of liver cancer varies significantly by sex and geographic region due to varying risk factors.^2^ These risk factors include infections such as hepatitis B virus, hepatitis C virus, and liver flukes in endemic areas, as well as behavioral factors such as alcohol and tobacco use, metabolic factors such as excess body fat, and exposure to aflatoxins.^3^ As the global community grappled with these increasing cancer concerns, pancreatic cancer emerged as a growing threat. By 2050, it is projected to have an incidence rate of 18.6 cases per 100 000, experiencing an annual increase of 1.1%.^4^ Despite advances in medical science, pancreatic cancer stubbornly held its position as the seventh leading cause of cancer-related mortality.^5^

Phytochemical substances, also known as secondary metabolites, are considered to be very promising because of their diverse biological and medicinal applications. They remain a key source of innovation in drug discovery, contributing to understanding essential biological pathways crucial for the drug development process.^6–8^ Exploring naturally occurring compounds in plants has led to the development of promising anticancer drugs. Cutting-edge technologies such as nanoparticle-based medicines are being used to enhance these drugs by allowing controlled drug release and innovative administration methods.^9,10^ In recent years, herbal medicines have gained attention in cancer treatment and prevention due to concerns about the toxicity of conventional chemotherapy drugs. Many medicinal plant extracts and phytochemicals show anticancer properties and around 60% of drugs are derived from natural sources, primarily medicinal plants.^11^ Plant-based phytochemicals exhibit a wide range of mechanisms that collectively contribute to inhibiting cancer development. They achieve this by neutralizing harmful free radicals, impeding the survival and growth of malignant cells, and reducing tumor invasiveness and angiogenesis of tumors.^12,13^ Furthermore, these metabolites have various and intricate effects on various molecular targets and signal transduction pathways, including membrane receptors, kinases, proteins that activate or suppress tumors, transcription factors, microRNAs, cyclins, and caspases.^14^ These phytochemicals further induce apoptosis, a programmed cell death process, by activating pro-apoptotic proteins and mitigating anti-apoptotic ones, while simultaneously modulating the expression of genes associated with apoptosis.^15^ Paclitaxel and resveratrol are the most common plant-based anticancer drugs approved for cancer treatment.^16,17^ Paclitaxel, originally derived from the Pacific yew tree, is a crucial anticancer drug used in the treatment of breast, ovarian, lung, and pancreatic cancers. It works as a microtubule-stabilizing agent, disrupting cell division, and inhibiting cancer cell proliferation by preventing the breakdown of microtubules. Often administered in combination with other anticancer drugs, paclitaxel has significantly improved cancer treatment outcomes. In the case of pancreatic cancer, it is used in conjunction with drugs such as gemcitabine to interfere with microtubules, disrupting the cell cycle and leading to cell death. This mechanism makes paclitaxel effective even in the challenging context of pancreatic cancer, slowing disease progression and enhancing overall treatment results.^18,19^ Resveratrol, a natural polyphenol found in various plants, including grapes and red wine, has been extensively investigated for its potential in cancer treatment.^20^ Resveratrol has been shown to inhibit the growth of various tumor cells, such as those in the breast, colon, prostate, liver, and lung. It also reduces tumor growth and metastasis in mice with highly metastatic lung carcinoma. These effects are attributed to the ability of resveratrol to impede DNA synthesis and inhibit neovascularization, and angiogenesis.^21,22^ In a mammary cancer model induced by DMBA, resveratrol reduces tumor incidence and multiplicity while extending the latency period. It also suppresses the activation of nuclear factor-κB, a regulator of genes associated with cancer development, including cyclooxygenase-2 and matrix metalloproteinase-9.^23^

*Cichorium endivia*, a perennial herb widely found in temperate regions, occupies a significant place in traditional healing systems such as Unani, Ayurveda, and Siddha.^24^ Different components of the plant have been used as traditional remedies worldwide. However, it should be noted that the root, in particular, harbors a plethora of significant phytochemical compounds.^25^ It is traditionally applied to address issues related to the renal system, hepatobiliary system, anorexia, and dyspepsia.^26,27^ Furthermore, beyond its medicinal applications, *Cichorium intybus* is used as an appetizer and in the treatment of conditions such as jaundice, liver failure, intermittent fever, and various skin disorders.^28^ Despite its extensive distribution, many of its constituents remain unexplored for their potential pharmacological benefits, and currently there are limited toxicological data available for the *Cichorium intybus* supplement.^29^

To thoroughly investigate the varied chemical classes, properties, and the extensive spectrum of metabolite concentrations present in plants, it is imperative to employ a diverse array of analytical techniques. Among the techniques, LC-MS/MS spectrometry has been used successfully and potentially for the exploration of the metabolic profile of various plant-derived extracts.^30^ In recent years, untargeted metabolomics has become a valuable technique for comprehensive analysis of a wide range of compounds within a single plant extract.^31^ This approach involves the simultaneous quantification of multiple low molecular weight compounds using techniques such as ultra-performance liquid chromatography (UPLC) coupled with mass spectrometry (MS).^32^ The application of advanced liquid chromatography tandem mass spectrometry (LC-MS/MS), distinguished by its exceptional resolution, has allowed a thorough exploration of the metabolome in various biological samples, including those from herbal medicine.^33^ Through the analysis of mass fragmentation (MS/MS) spectra, valuable insights into the structural characteristics of metabolites can be inferred from their unique spectral patterns.^34^ Metabolomics is extensively applied in studies of plant metabolism to explore the plant system at the molecular level and furnish a characterization of the complete metabolite pool within a plant tissue. This approach yields substantial information about chemical species exhibiting diverse physical properties.^35^ Untargeted metabolomics, which encompasses the comprehensive and unbiased measurement of all metabolites within a plant extract, provides a broader perspective on the fundamental biochemical processes occurring within that particular plant extract.^36^ In light of these facts and our keen interest in deciphering new pharmacological potential agents,^37–43^ we aimed in this study to reveal the phytochemical profile of the ethanolic extract of *Cichorium intybus* roots (CIR) and to explore its antiproliferative potency in a set of 15 cell lines. In this regard, we employed various analytical and chemical techniques, including UPLC/T-TOF-MS/MS-based analysis, to deep explore the phytochemical profile of the CIR extract. We further screened the cytotoxic activity of the CIR extract in a panel of cancer cells and performed a comprehensive analysis to reveal the mode of action of the antiproliferative activity by assessing programmed cell death, cell cycle arrest, and apoptotic-related genes. Furthermore, due to the fact that cyclooxygenases play a crucial role in inflammatory conditions, including cancer initiation and progress,^44,45^ we evaluated the antiproliferation activity of CIR extract by evaluating the inflammatory signaling of cyclooxygenase 1 & 2 and a subset of downstream targets such as the IL-1b, IL-6 and TNF-α genes.^46^ Finally, the antioxidant activity of the CIR extract was explored by evaluating not only the total antioxidant capacity, but also its free radical and metal scavenging activity.

## 2 Materials and method

### 2.1. Materials

Analytical grade solvents, including acetonitrile, ethyl acetate, methanol, ethanol, dichloromethane, and isopropanol, were obtained from Thermo-Fisher. Reagents including ammonium hydroxide, MTT, formic acid, DMSO, ammonium acetate, trypan blue dye, and ammonium formate were sourced from Sigma-Aldrich. Cell culture reagents, including fetal bovine serum, DMEM, RPMI-1640, HEPES buffer solution, l-glutamine and gentamycin, were purchased from Lonza (Belgium).

### 2.2. Plant collection and extraction method

*Cichorium endivia* L. *divaricatum*, synoun (*Cichorium inytubs* L.), family Asteraceae is an annual winter plant indigenous to the Mediterranean region. Species were collected in March 2023 from cultivated fields in Egypt, particularly sourced from fields of *Trifolium alexandrinum* (Egyptian clover or berseem clover). Plant roots were cleansed with distilled water, lyophilized and pulverized using a mixer mill (Retsch MM400, Germany) before the extraction process. Taxonomic identification was conducted according to the methodology outlined by Boulos L. in “*Flora of Egypt*, vol. 1–4” (1999–2005), published by Al Hadara Publishing in Cairo, Egypt. The samples were deposited in the Alexandria Herbarium for preservation and reference. Five hundred grams of powdered roots were mixed with 70% ethanol (EtOH) or deionized water (240 mL) and were extracted for 3 days on a Kuhner IRC-1-U rotary shaker (Clim-O-Shake system Kuhner IRC-1-U) at 28 °C. The resulting extract mixture was double filtered using filter paper under vacuum and then the collected filtrate was dried using a rotary evaporator to yield the dry extract as waxy oil. The crude extract was finally lyophilized to provide the ethanolic CIR extract as a powder which was kept at −5 °C until utilization.^47,48^

### 2.3. Chemical characterization

#### 2.3.1. Total nutrients

Total nutrients in CIR extract were assessed by conducting various reported analyzes, including total lipid content (conventional assay), total saponin content (modified vanillin–sulphuric acid assay), total carbohydrate content (phenol–sulphuric acid assay), total protein content (Bradford assay), total alkaloid content (bromocresol green-based colorimetric assay), total phenolic content (Folin–Ciocalteu assay), total flavonoid content (aluminum chloride colorimetric assay) and total tannin content (acidified-vanillin assay).^49^

#### 2.3.2. Ultra-performance liquid chromatography mass spectrometry analysis (UPLC/T-TOF-MS/MS)

In this study, the UHPLC/QTOF-MS technique was used to determine the metabolic profile of the CIR extract.^50,51^ Briefly, the lyophilized CIR extract (50 mg) was dissolved in a 1 mL solution mixture of MeOH : ACN (2 : 1 ratio, v/v), and subsequently subjected to a vortex mixing, ultrasonication, and centrifugal to provide a transparent supernatant of concentration of 1.0 μg mL^−1^ concentration. The obtained sample was injected (10 μL) into HPLC/QTOF-MS/MS to be analyzed in positive and negative electrospray ionization (ESI, at +4500/+80 V and 4500/80 V) modes using a C18-RP column with (2.1 × 150 mm, 2.5 μm particle size, Waters, XSelect HSS T3, Milford, MA, USA). The mass spectrometer functioned by conducting a 50 ms survey scan (TOF-MS), followed by automatic selection of the top 20 ions for subsequent fragmentation spectra. The mass range covered during the analysis ranged from 50 to 1100 *m*/*z*. Implementing a gradient elution strategy at 0.3 mL min^−1^, the mobile phase consisted of two components. Part A, a mixture of 5 mM ammonium formate solution in water with 1% (v/v) methanol, adjusted to pH 3.0, and part B, is composed of HPLC grade acetonitrile. The elution gradient started with 10% part B up to 20 minutes, increased to 90% up to 25 minutes, and then reverted to 10% up to 28 minutes. Data were gathered through IDA (AB SCIEX analyst TF 1.7.1). Multivariate data analysis for nontargeted metabolomic analysis was performed comprehensively utilizing MS-DIAL (3.70 Yokohama, Kanagawa 230-0045 software, Japan), along with Peak View software from SCIEX.^39,50,51^

### 2.4. Assessment of antiproliferative activity

The cytotoxic activity of the CIR root extract was assessed for a panel of cell lines, including HepG-2 cells (human pancreatic cancer cell line), HCT-116 cells (human colon cancer cell line), MCF-7 cells (human breast cancer cell line), MDA-MB-231 cells (human breast cancer cell line), PC-3 cells (human prostate cancer cell line), A-549 (human lung cancer), A-431 cells (human skin cancer cell line), HeLa cells (human cervical cancer), RD cells (human muscle rhabdomyosarcoma cancer cell line), Panc-1 cells (human pancreatic cancer cell line), CACO2 cells (human intestinal cancer cell line), HEp-2 cells (human larynx cancer), SKOV-3 cells (human ovarian adenocarcinoma), MRC-5 cells (normal human lung fibroblast cells) and SAOS-2 cells (human osteosarcoma). These cell lines were obtained from (the Regional Center for Mycology and Biotechnology at Al-Azhar University). Cells were propagated in specific growth medium and maintained under standard conditions at a temperature of 37 °C in a well-humidified environment with 5% carbon dioxide with regular subculture. Evaluation of antiproliferative activity was carried out following the protocol previously reported.^42,43,52^ Briefly, 96-well plates were utilized for cell seeding. After a 24 hours incubation, CIR was introduced at varying concentrations. Control wells treated with only DMSO were also included. Following another 24 hours incubation, the evaluation of viable cell numbers was conducted using the MTT assay. In particular, the culture medium was substituted with an MTT reagent and plates were incubated. Subsequently, DMSO was introduced and the optical density was measured at 590 nm with the microplate reader. The relation between surviving cells and CIR concentration was plotted using GraphPad Prism software to obtain the survival curve of each cell line and the IC50 value was derived from a graph.

### 2.5. Microscopic assessment of tumor cell morphology

After treatment of cells with CIR extract at the designated concentration, the medium was drained by inverting the plate and washed with pH 7.2 phosphate buffered saline. Subsequently, cells were immobilized on the plate by exposure to 10% formalin for 15 minutes at room temperature. Cells underwent the staining process using 100 μL of 0.25% crystal violet for a duration of 20 minutes. After the staining process, the excess dye was eliminated, and the plates underwent a deionized water before air drying. Using an inverted microscope (CKX41; Olympus, Japan), images were taken to assess cellular morphology and detect changes compared to control cells. Microscopic examination at 100× magnification facilitated the observation of cytopathic effects or morphological variations.

### 2.6. Evaluation of cell cycle arrest

Cell cycle analysis was performed using the propidium iodide (PI) flow cytometry kit (ab139418, Abcam, USA) by measuring the DNA content using a flow cytometer following the manufacturer's instructions.^43,53,54^ Briefly, the CIR was dispersed in the cells, centrifugated at 500 g for 5 minutes, washed with 1× PBS, fixed with 66% ethanol on ice and resuspension of the cell pellet in 1× PBS followed by 100% ethanol. The subsequent PI staining procedure involved equilibrating the cells at room temperature, washing with 1× PBS and resuspending the cells in 1× PI + RNase staining solution. After incubation for 1 h in the dark at 37 °C, cells were analyzed using a flow cytometer with an excitation at 493 nm and an emission at 636 nm.

### 2.7. Annexin V analysis for the evaluation of apoptosis

The Annexin V detection method was utilized to assess the apoptosis and necrosis activity of the CIR extract following previous reports.^53^ Briefly, cells undergo incubation with Annexin V-FITC to stain apoptotic cells, collected by centrifugation and resuspended in 500 μL of 1× binding buffer. Subsequently, 5 μL of Annexin V-FITC and 5 μL of propidium iodide (PI 50 mg mL^−1^) were added and incubated at room temperature for 5 minutes in the dark. The quantification of Annexin V was subsequently analyzed by flow cytometry through the FITC signal detector, while PI staining was detected *via* the phycoerythrin emission signal detector (usually FL2).

### 2.8. Evaluation of apoptotic and inflammatory-related gene expression

We evaluated the expression of key genes related to apoptosis and inflammation, including Bcl-2-associated X protein (Bax), B cell lymphoma 2 (BCl2), tumor protein P53 (P53), Caspase-3 (Casp3), cytochrome C (CYC), TNFα, IL1B, and IL6 (Table S1†). This examination was carried out on HepG2 and Panc-1 cells after a treatment period lasting two days with CIR extract at 37 °C compared to staurosporine or untreated control cells. RNA extraction and purification were conducted using the Qiagen RNeasy Mini Kit manufactured by (Hilden, Germany). Subsequently, 1 μg of RNA was used for cDNA synthesis using the cDNA kit (Sigma-Aldrich, product no. 11117831001). Real-time PCR measurements were performed using the Thermo Fisher Quantstudio 5 0.1 ML system and the iScript TM SYBR® Green One-Step RT-PCR Kit was used (Bio-Rad, Hercules, California, United States).^55–58^ Briefly, 10 ng of cDNA was used and the cycling conditions consisted of an initial heating stage for 5 minutes at 95 °C, followed by 45 cycles of denaturation at 95 °C for 10 seconds, annealing at 60 °C for 30 seconds and extension at 72 °C for 1 minute. Subsequently, the relative gene expression was assessed using the 2^−ΔΔCT^ equation, with the data normalized to the reference gene GAPDH.

### 2.9. Evaluation of antioxidant activity

#### 2.9.1. Free radical DPPH scavenging assay

According to Valko *et al.*,^59^ the antioxidant capacity of CIR root extract was evaluated using a free radical DPPH scavenging assay. Briefly, CIR extract at different concentrations (0.5–1000 μg mL^−1^) was mixed with the DPPH solution (0.66 mM) and incubated at 25 °C for 20 minutes. After incubation, the absorbance was measured at 510 nm using a spectrophotometer. For comparison purposes, ascorbic acid was used as the standard reference compound. The IC_50_ value was determined from the inhibition concentration curve using the GraphPad program.

#### 2.9.2. Evaluation of H_2_O_2_ scavenging activity

The evaluation was carried out according to the protocol described by Ruch *et al.*^60^ Briefly, a CIR extract or ascorbic acid (reference) solution at different concentrations (0.5 to 1000 μg mL^−1^) was treated with a hydrogen peroxide solution in phosphate buffer (0.6 mL, 40 mM, pH 7.4). After incubation of the resulting solution for 30 min, the absorbance of the solution was subsequently examined at 230 nm using a spectrophotometer. The IC_50_ value was determined from the inhibition concentration curve using the GraphPad program.

#### 2.9.3. Evaluation of ABTS scavenging activity

The scavenging activity of the CIR extract against the ABTS radical was evaluated following the reported protocol with minor modifications.^61^ Briefly, a solution of 7 mM ABTS (5 mL) was treated with potassium persulfate (88 μL, 140 mM), and the resulting solution was incubated in the dark overnight. Subsequently, CIR root extract (0.5–1000 μg mL^−1^) was introduced to 100 μL of ABTS solution, and the mixture was incubated for 30 min to react. Finally, the absorbance was measured at 734 nm using a spectrophotometer. In our analysis, ascorbic acid was used as a control in this assay. The IC_50_ value was determined from the inhibition concentration curve using the GraphPad program.

#### 2.9.4. Assessment of metal chelation activity

Metal chelating activity was evaluated utilizing the reported protocol.^62^ Briefly, CIR root extract (0.2 mL) at different concentrations (0.5 to 1000 μg mL^−1^) was mixed with a solution of ferrozine (0.25 mM, 0.4 mL) and FeSO_4_ (0.1 mM, 0.2 mL). After incubation of the mixture at 27 °C for 1 h, the absorbance was assessed using a 562 nm spectrophotometer. The IC_50_ value was determined from the inhibition concentration curve using the GraphPad program. In our analysis, EDTA was employed as a control in this assay.

#### 2.9.5. FRAP assay

The Ferric Reducing Antioxidant Power (FRAP) assay was carried out following an established protocol with minor modifications.^29^ Briefly, the FRAP reagent was mixed by mixing (2,4,6-tripyridyl-*s*-triazine) (2.5 mL, 10 mM in HCl), hydrated FeCl_3_ (2.5 mL, 10 mM) in acetate buffer (300 mM, pH 3.6). After the FRAP solution was heated at 37 °C, it was mixed with CIR extract at different concentrations (0.5 to 1000 μg mL^−1^). The resulting mixture was incubated for 1 h under the same conditions before absorbance was examined using a spectrophotometer at 593 nm. FRAP activity was expressed as FeSO_4_ (μM) per gram of CIR root extract. In our analysis, ascorbic acid was used as a control in this assay. The IC_50_ value was determined from the inhibition concentration curve using the GraphPad program.

#### 2.9.6. Cupric reducing antioxidant capacity assay (CUPRAC)

The CUPRAC assay was conducted following the protocol reported protocol by Apak *et al.*^63^ Briefly, the CUPRAC solution was freshly prepared by (1 : 1) mixing copper(ii) chloride (10 mM) and neocuproine solution (7.5 mM) with ammonium acetate buffer solution (1 M, pH 7.0). Subsequently, CIR extract at different concentrations (0.2 mL, 0.5 to 1000 μg mL^−1^) was introduced to the CUPRAC solution and the resulting mixture was incubated for 1 h at room temperature. Finally, CUPRAC activity was evaluated by examining the absorbance by a 450 nm spectrophotometer. In our analysis, EDTA was employed as a control in this assay. The antioxidant activity was then determined and expressed as EDTA equivalents per gram of the CIR extract. The IC_50_ value was determined from the inhibition concentration curve using the GraphPad program.

#### 2.9.7. Assessment of total antioxidant capacity (TAC)

Total antioxidant capacity (TAC) was examined following the protocol of Prieto *et al.*^64^ The TAC solution was prepared by mixing ammonium molybdate (1 g, 28 nM) and sodium sulfate (1.24 g, 4 mM) in water (250 mL). In the TAC solution (3 mL), CIR extract (300 μL) was introduced and the resulting mixture was incubated at 90 °C for 2 h. After the mixture was cooled to ambient temperature, the absorbance was evaluated using a 695 nm spectrophotometer. The TAC was expressed as milligrams of gallic acid equivalents (GAE) per gram of CIR extract.

### 2.10. Evaluation of anti-inflammatory activity

#### 2.10.1. Evaluation of cyclooxygenase 1 (COX-1) activity

The evaluation of the inhibitory activity of CIR extract with respect to COX-1 activity was used following the reported procedure.^7^ In this analysis, ibuprofen served as a control. Briefly, the COX-1 solution, sourced from soybean, was treated with CIR root extract or ibuprofen at varied concentrations for a duration of 15 minutes at room temperature. Subsequently, the mixture was treated with arachidonic acid/NaOH solution and the absorbance was assessed using a microplate reader at 234 nm. The determination of the IC_50_ value was made from the concentration-dependent curve using the GraphPad program.

#### 2.10.2. Evaluation of cyclooxygenase 2 (COX-2) activity

The inhibitory potential of the CIR root extract for COX-2 activity was examined and compared to ibuprofen following reported procedure.^7^ Briefly, CIR extract or ibuprofen was incubated at different concentrations in a mixture of *N*,*N*,*N*,*N*-tetramethyl-*p*-phenylenediamine, arachidonic acid, and COX2 enzyme (Cat.#K547). The inhibitory impact was assessed using a microplate reader at 611 nm absorbance. IC_50_ values were calculated from the concentration-dependent curve using the GraphPad program.

### 2.11. Molecular modeling study

The molecular coupling study presented was conducted using Molecular Operating Environment (MOE, 2015.10) software to conduct the molecular coupling study of selected secondary metabolites detected in the CIR extract towards the binding pocket of cyclooxygenase 1 & 2 (COX1 & 2) proteins. Toward this end, the crystal structure of the targeted proteins was acquired from the protein database (COX-1: PDB ID 1eqg, COX-2 PDB ID: 4ph9).^65,66^ The crystal structures were carefully checked and selected on the basis of the nature of the cocrystallized ligand. In our study, we selected crystal structures that cocrystallized with ibuprofen as a reference drug. The acquired crystal structures were initially adjusted by deleting additional chains and water molecules and protonating the protein using the default protocol. The structure of selected secondary metabolites was acquired from the ChemDraw program, and energy minimization and geometry verification were assessed using the MOE program as previously reported.^53,54,67–70^ The modelling protocol was adjusted for the force field (MMFF94x), the scoring function (London dG), and the placement (Triangle) and subsequently validated by modelling the binding mode of ibuprofen. The binding poses were then evaluated to affirm the original binding mode of ibuprofen to the target proteins, compared to the reported data. The adjusted methodology was then used to explore the binding mode and affinity of the selected secondary metabolites of the CIR extract toward the selected COX proteins. The collected data was analyzed and visualized using the MOE program to provide the binding affinity (kcal mol^−1^) and binding mode of the examined metabolites.

### 2.12. Statistical analysis

Statistical analysis was performed using GraphPad Prism version 9 software (GraphPad Software, San Diego, CA, USA). Data are presented as mean ± S. D. The Student's *t*-test was used for the comparison of two groups. The ANOVA test was used for multiple group comparison followed by the Tukey test as a *post hoc*. The differences between the groups were considered significant when *p* < 0.05 (*p < 0.05, **p < 0.01, ***p < 0.001 and * ***p < 0.0001).

## 3 Results and discussion

### 3.1. Phytochemical characterization

#### 3.1.1. Total nutrient analysis

We initially evaluated the total nutrients in the CIR extract, including saponin, ash, tannins, proteins, carbohydrates, lipids, phenolics, alkaloids, and flavonoids, after the chemical and colorimetric analysis previously reported. As indicated in Table S2,† our analysis revealed that CIR has a substantial content of flavonoids, phenolics, and proteins with 106.5, 55.71, and 48.11 mg mL^−1^, respectively. Furthermore, our findings indicated that the CIR extract possesses a considerable content of saponin, lipids, and tannins. This indicates the potential of CIR extract to serve as a source of beneficial metabolites with various biological activities, such as antioxidant and anti-inflammatory effects. However, the CIR extract exhibited low concentrations of ash, alkaloids, and carbohydrates. Although the ash content provides information on the mineral composition of the extract, alkaloids and carbohydrates are valuable nutrients. Together, these results indicate that the CIR extract possesses a diverse nutrient profile that underscores its therapeutic potential in various applications, including nutraceuticals and pharmaceuticals.

#### 3.1.2. Untargeted metabolomic analysis (UPLC/T-TOF-MS/MS)

**Fig. 1:**
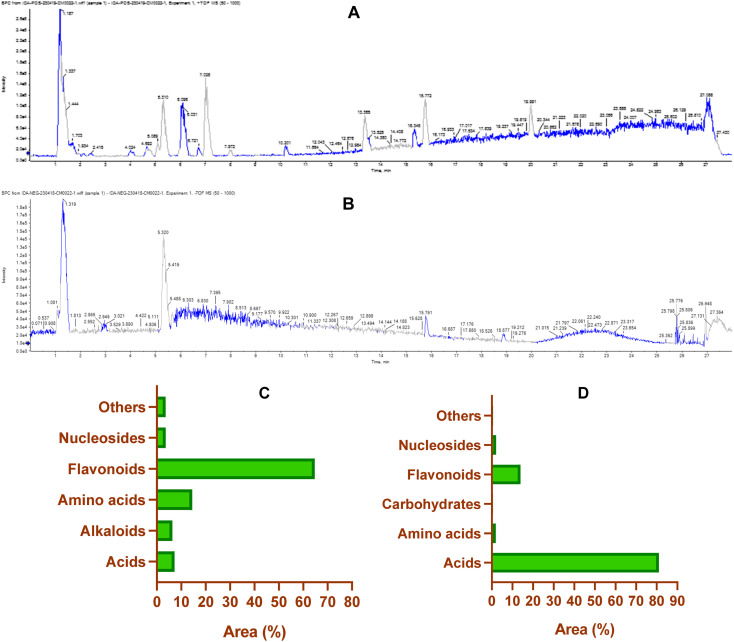
The base peak chromatogram of CIR extract in the positive (A) and negative (B) electrospray ionization modes by using UPLC/T-TOF-MS/MS analysis. Representative graphs describing the classification of the detected primary metabolites (% of the total area) in the positive (−) ESI (C) and negative (−) ESI (D) ionization modes.

The non-targeted metabolomic approach involves a comprehensive exploration and detection of diverse bioactive metabolites within plant extracts.^71–73^ In the present study, ultra-performance liquid chromatography-mass spectrometry (UPLC/T-TOF-MS/MS) was employed to enable phytochemical profiling of CIR extract in both positive and negative ionization modes. As detailed in Tables S3 and S4,† the LCMS/MS analysis revealed 131 metabolites in the CIR extract. The 131 detected metabolites in the CIR extract were classified into various classes, including acids,^26^ amino acids,^19^ flavonoids,^35^ alkaloids,^2^ nucleotides,^20^ carbohydrates,^4^ and a set of miscellaneous metabolites.^25^ The positive ionization mode revealed a set of 72 metabolites that belong to five distinct classes: flavonoids (20 metabolites, 64.7% peak area), amino acids (12 metabolites, 14.5% peak area), acids (12 metabolites, 7.2% peak area), nucleotides (9 metabolites, 3.6% peak area), alkaloids (2 metabolites, 6.3% peak area) and miscellaneous metabolites (19 metabolites, 3.6% peak area) (Table S3†). Beyond flavonoids, two subgroups were distinguished: one comprised of 7 flavonoid metabolites and the other consisting of 13 flavonoid-*O*-glycosides. Among flavonoids, three specific flavonoids emerged as the most prevalent metabolites: 3 4 5 7-tetrahydroxyflavanone (16.02% peak area), naringenin (9.8% peak area) and 3,5,7-trihydroxy-4′-methoxyflavone (6.5% peak area). In flavonoid *O*-glycosides, acacetin-7-*O*-rutinoside (26.2% peak area) emerged as the predominant metabolites detected in positive ionization mode, with isorhamnetin-3-*O*-glucoside (2.2% peak area) displaying a discernible presence. Regarding amino acid metabolites, 12 distinct metabolites were observed, which constitute approximately 14.5% of the detected metabolites in positive ionization mode. *N*,*N*-Dimethylglycine was the most prevalent amino acid metabolite (9.4% peak area), followed by dl-5-hydroxylysine (1.4% peak area). Acid-based metabolites constituted around 7.2% peak area (10 metabolites) of the total identified metabolites, with 12-oxo-10,15(*Z*)-phytodienoic acid (4% peak area) and *trans*-cinnamate (2.6% peak area) being the most predominant acid-based metabolites. Furthermore, our analysis identified two alkaloid metabolites of considerable predominance, trigonelline (3.4% peak area) and harmaline (3.0% peak area). Our analysis also revealed a considerable concentration of nucleotide metabolites (nucleobase), with a total peak area of 3.6% (9 metabolites). 2-Deoxycytidine was the most prominent metabolite in this class with a peak area of 1.72%. The further analysis encompassed a category of miscellaneous metabolites (19 metabolites) that contributed to a cumulative concentration of 3.6% total peak area. Within this subset, glycerol-2-phosphate (0.9% peak area) and 4-aminophenol (0.8% peak area) displayed notable abundance (Fig. 1).

On the other hand, the LCMS/MS analysis of the CIR root extract in negative ionization mode successfully detected 59 metabolites (Table S4†), spanning six diverse classes, namely acids (16 metabolites, 80% peak area), amino acids (7 metabolites, 1.89% peak area), carbohydrates (4 metabolites, 0.5% peak area), flavonoids (15 metabolites, 14% peak area), nucleosides (11 metabolites, 2.1% peak area) and other miscellaneous metabolites (6 metabolites, 0.6% peak area). Within the category of flavonoids, 3 metabolites belonged to flavonoids, while 12 metabolites belonged to flavonoid-*O*-glycosides. Apigenin emerged as the predominant metabolite in this class, contributing 8.4% of the total peak area, while peonidine-3-*O*-glucoside chloride exhibited a total peak area of 1.5%. Our findings emphasized that more than 80% of the peak area of the identified metabolites falls within the acid-based class, with 16 distinct metabolites detected. Remarkably, γ-linolenic acid led this category with 58.9% of the total peak area, followed by gluconate (8.2% peak area), d-(−)-quinic acid (5.4% peak area) and d-(+)-malic acid (5.1% peak area). Nucleotide-based metabolites were detected with approximately 2.1% of the total peak area. Uridine, as the most predominant metabolite within this category, represented 0.7% of the total peak area. Furthermore, amino acid metabolites constituted 1.9% of the total detected metabolites in the negative ionization mode. Notably, l-γ-homoisoleucine emerged as the most prevalent amino acid, accounting for 0.9% (peak area). Our analysis further revealed that CIR extract possessed considerable carbohydrate-based metabolites (0.5% peak area, 4 metabolites). Finally, a group of six miscellaneous metabolites collectively account for 0.6% of the peak area. Among these metabolites, sodium deoxycholate was the most abundant, with a detection of 0.25% (peak area).

**Fig. 2:**
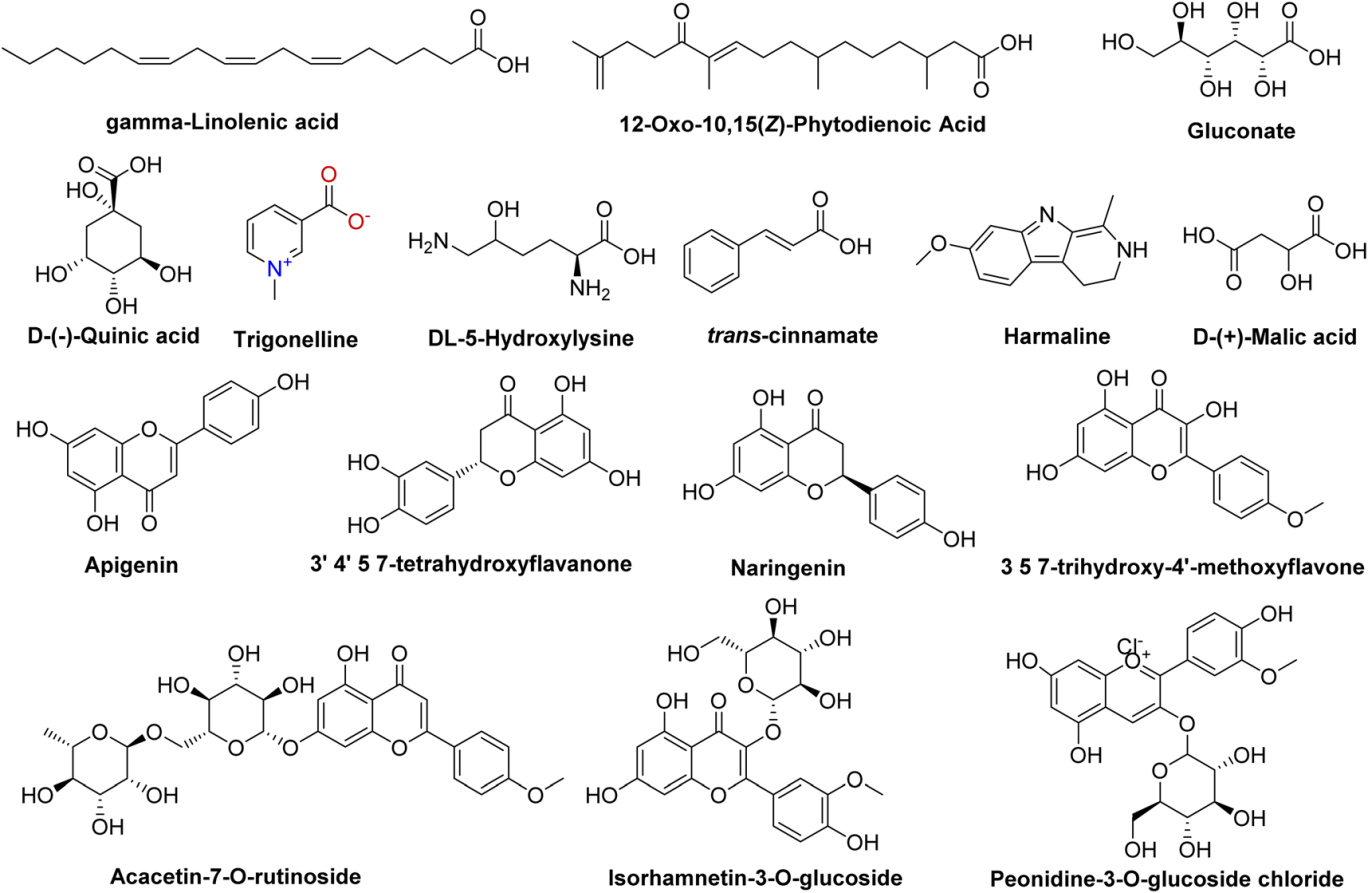
Representative structures of the most prominent metabolites detected in the CIR extract by UPLC/T-TOF-MS/MS analysis in positive and negative ionization modes.

Our comprehensive profiling of the CIR root extract illuminated the intricate metabolite composition inherent in this extract. The most prominent metabolites (59-1% peak area) identified by untargeted metabolomic analysis of CIR extract are detailed in Table 1 and Fig. 2. Consequently, CIR extract emerges as a promising candidate for natural supplementary interventions addressing a wide array of health issues. Our analysis indicated that the CIR extract possesses high concentrations of acid-based metabolites including γ-linolenic acid (GLA), gluconate, d-(−)-quinic acid, d-(+)-malic acid, and 12-oxo-10,15(*Z*)-phytodienoic acid. GLA was represented as the most prominent metabolite in this class and as a major constituent of the CIR extract. Several studies showed that GLA has the potential to mitigate conditions associated with chronic inflammation. Furthermore, GLAs showed an acknowledged role in maintaining skin health that extends to applications in skincare formulations. Furthermore, GLA contributes to the support of the immune system and cardiovascular well-being by regulating cholesterol levels.^74,75^ Gluconate, a naturally carboxylic acid, contributes significantly contributes to cellular metabolism^76^ and its biological impact extends to various applications, including anti-inflammatory, antioxidant, immunomodulatory, antimicrobial, and antiviral effects.^77,78^

**Table 1:** The major metabolites tentatively detected in the ethanolic extract of CIR extract using UPLC/T-TOF-MS/MS in positive and negative ionization modes

Title	RT (min)	Precursor (*m*/*z*)	Area	Error (PPM)	Adduct	Reference (*m*/*z*)	Formula	Ontology
Maleic acid	0.87	115.0034	637 716	−0.6	[M − H]^−^	115.00368	C_4_H_4_O_4_	Dicarboxylic acids
d-(+)-Malic acid	0.88	133.0128	3 334 704	6.6	[M − H]^−^	133.01425	C_4_H_6_O_5_	β-Hydroxy acids
d-(−)-Quinic acid	0.93	191.0543	3 485 385	7.4	[M − H]^−^	191.05611	C_7_H_12_O_6_	Quinic acids
Gluconate	0.96	195.0495	5 327 352	6	[M − H]^−^	195.05103	C_6_H_12_O_7_	Hydroxy acids
*N*,*N*-Dimethylglycine	1.053	104.1062	4 379 100	3.9	[M + H]^+^	104.0706	C_4_H_9_NO_2_	Amino acid
12-Oxo-10,15(*Z*)-phytodienoic acid	1.12	293.0635	1 886 257	−1.2	[M + H]^+^	293.21112	C_18_H_28_O_3_	Acid
Trigonelline	1.14	138.0552	1 573 969	−0.1	[M + H]^+^	138.05496	C_7_H_7_NO_2_	Alkaloids
dl-5-Hydroxylysine	1.18	163.058	673 048.7	12.8	[M + H]^+^	163.10771	C_6_H_14_N_2_O_3_	Amino acid
l-Asparagine	1.29	133.0507	512 949.9	−4.8	[M + H]^+^	133.06078	C_4_H_8_N_2_O_3_	Amino acid
Harmaline	5.06	215.1256	1 403 891	0.1	[M + H]^+^	215.11789	C_13_H_14_N_2_O	Alkaloids
3′,4′,5,7-Tetrahydroxyflavanone	5.27	289.163	7 469 832	0.8	[M + H]^+^	289.07068	C_15_H_12_O_6_	Flavonoids
Isorhamnetin-3-*O*-glucoside	6.79	479.12	1 024 418	0.8	[M + H]^+^	479.11841	C_22_H_22_O_12_	Flavonoids
Peonidine-3-*O*-glucoside chloride	7.39	461.1098	1 011 160	−0.8	[M − 2H]^−^	461.1084	C_22_H_23_O_11_	Anthocyanidin-3-*O*-glycosides
Naringenin	7.53	273.1644	4 594 841	13.8	[M + H]^+^	273.07574	C_15_H_12_O_5_	Flavonoids
Apigenin	10.40	269.0444	5 452 233	3.3	[M − H]^−^	269.04553	C_15_H_10_O_5_	Flavones
γ-Linolenic acid	19.07	277.2176	37 943 284	−0.9	[M − H]^−^	277.21732	C_18_H_30_O_2_	Lineolic acids
3,5,7-Trihydroxy-4′-methoxyflavone	19.66	301.1429	3 023 031	0.6	[M + H]^+^	301.07068	C_16_H_12_O_6_	Flavonoids
2′-Deoxycytidine	19.92	228.2302	800 974	12	[M + H]^+^	228.09789	C_9_H_13_N_3_O_4_	Nucleosides
*trans*-Cinnamate	22.58	149.0246	1 220 626	−1.9	[M + H]^+^	149.05971	C_9_H_8_O_2_	Acid
Acacetin-7-*O*-rutinoside	24.03	593.2783	12 237 822	0.1	[M + H]^+^	593.18646	C_28_H_32_O_14_	Flavonoids

d-(+)-Malic acid exhibits notable biological activities, mainly known for its hepatoprotective effects.^79^ Additionally, it plays a role in the regulation of acidity in the body and participates in the citric acid cycle, an essential metabolic pathway.^80^ Additionally, 12-oxo-10,15 (*Z*)-phytodienoic acid has been involved in the regulation of various physiological processes in plants, including defense responses.^81^ Finally, *trans*-cinnamate demonstrated a wide diversity of bioactivity including antioxidant, antimicrobial, and anti-inflammatory potentials, suggesting its potential to alleviate conditions associated with inflammation.^82^ Flavonoids and flavonoid *O*-glycoside metabolites have also been detected in substantial concentrations, including acacetin-7-*O*-rutinoside, 3′,4′,5,7-tetrahydroxy-flavanone, naringenin, apigenin, 3,5,7-trihydroxy-4′-methoxyflavone, peonidine-3-*O*-glucoside and isorhamnetin-3-*O*-glucoside. Acacetin-7-*O*-rutinoside, the second highly detected metabolite, plays a crucial role in various biological activities, including anti-inflammatory, antimicrobial, and anticancer properties.^83,84^ Acacetin also demonstrates a range of biological activities, emerging as a potential agent for improving insulin sensitivity and regulating blood sugar levels, thus promising in the management of diabetes.^85^ Similarly, 3′,4′,5,7-tetrahydroxyflavanone, a naturally occurring flavone, is characterized by its anticarcinogenic, anti-inflammatory and antioxidant capabilities, but also offers cardiovascular protection and stimulates the immune response.^86,87^ The CIR extract also exhibited a notable abundance of naringenin, which encompasses a diverse array of therapeutic potential, including antioxidant, anti-inflammatory, anti-apoptotic, anti-ulcer, anti-osteoporotic, and anti-carcinogenic effects.^88,89^ Extensive research has empirically supported its pharmacological benefits as a multifaceted agent with hepatoprotective, antiatherogenic, antimutagenic and antimicrobial properties.^90,91^ Peonidine-3-*O*-glucoside chloride, a flavonoid glycoside detected in CIR extract, holds promise as antioxidant activity, contributing to cell protection against oxidative stress.^92^ Apigenin, another detected flavonoid, possesses various bioactive properties, including antibacterial, antiviral, anti-inflammatory, and anticancer activities.^93–95^ Its versatile pharmacological profile involves the modulation of signaling pathways and cellular processes, making it a promising natural compound for potential therapeutic applications, including rheumatoid arthritis, autoimmune disorders, and neurodegenerative diseases.^96^ Relatedly, 3,5,7-trihydroxy-4′-methoxyflavone has been recognized for its potential pharmacological applications as an anticancer, anti-inflammatory, and antioxidant.^97^ LCMS-MS analysis also revealed a considerable abundance of amino acid metabolites including *N*,*N*-dimethylglycine, l-asparagine, and 5-hydroxylysine. *N*,*N*-Dimethylglycine, commonly denoted as vitamin B16, has versatile therapeutic applications that include the treatment of depression in middle-aged and elderly individuals,^98^ the facilitation of the human immune system,^99^ and the mitigation of cholesterol levels within the body.^100^ Although dl-5-hydroxylysine has been implicated in collagen formation,^101^l-asparagine plays diverse roles in protein synthesis, neurotransmitter regulation, and cellular energy metabolism.^102,103^ Two alkaloids, trigonelline and harmaline, were also identified in the CIR root extract in remarkable abundance. Trigonelline has potential antioxidant, neuroprotective and anti-inflammatory properties and has been implicated in the treatment of diabetes through improved glucose metabolism.^104,105^ On the other hand, harmaline exhibited substantial neuropharmacological and antiamnesic effects. In addition, studies demonstrated its ability to improve cognition dysfunction associated with neurodegenerative diseases.^106,107^ Collectively, comprehensive metabolomic analysis underscores the potential of the CIR extract as a valuable source of bioactive compounds with promising applications in the treatment of various health issues. Aligning with our findings, previous investigations revealed that the extract of *C. intybus* exhibits a set of unique metabolites that include guaianolides (*e.g.*, lactucin, lactucopicrin), polyacetylenes (*e.g.* putrescine, spermidine) and phenolic compounds (*e.g.*, chlorogenic acid, caffeic acid), flavonoids (*e.g.*, apigenin-7-*O*-glucoside, kaempferol-7-*O*-glucoside, quercetin-3-*O*-glucoside) and sterols (*e.g.*, β-sitosterol, campesterol, stigmasterol). The phytochemical diversity of *C. intybus* further underscores its potential as a source of bioactive metabolites with miscellaneous therapeutical effects.^29^ Another investigation of the phenolic metabolites of red *C. intybus* utilizing high-performance liquid chromatography analysis (HPLC-DAD-ESI-MS/MS) identified malic acid and quinic acid among the free small organic acids, various caffeic acid derivatives, and different isomers of caffeoylquinic acids. Furthermore, various derivatives of kaempferol, which include mono and diglycosides, along with other flavonols such as isorhamnetin, quercetin, and myricetin derivatives, were also characterized based on their MS fragmentation patterns.^108^

### 3.2. Assessment of cytotoxic activity

**Fig. 3:**
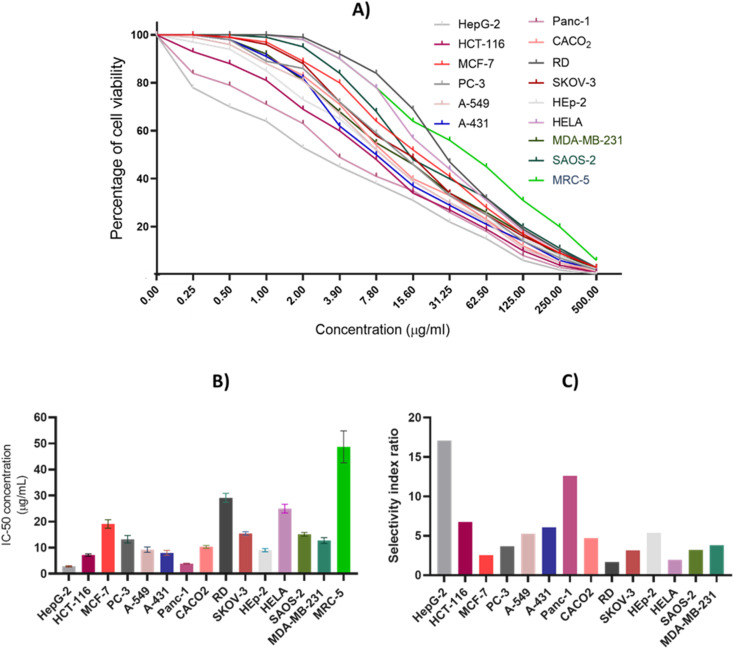
The antiproliferative effect of CIR extract on the cellular viability of a panel of 15 human cell lines. (A) Dose-dependent cytotoxic effect of CIR extract on the viability of examined cells. (B) IC_50_ (μg mL^−1^) values of the CIR extract in relation to the examined cells. (C) The selectivity of the cytotoxic effect of the CIR extract toward the examined cancer cell lines, compared to that of MRC-5 cells. Data were expressed as mean ± S. D in triplicate.

A comprehensive antiproliferative assay was conducted to detect and evaluate the cytotoxic impact of CIR extract against a panel of 15 human cell lines including HepG-2, HCT-116, MCF-7, PC-3, A-549, A-431, Panc-1, CACO2, RD, SKOV-3, HEp-2, HELA, SAOS-2, MDA-MB-231, and MRC-5. The CIR extract was assessed dose-dependently, with concentrations ranging from 0.25 to 500 μg mL^−1^, and cell viability was examined by MTT assay utilizing a spectrophotometer. As depicted in Fig. 3, our results showed that the CIR extract exhibits a remarkable dose-dependent cytotoxic activity in all cancer cell lines examined with IC_50_ values 2.85–29.15 μg mL^−1^. While the extract exhibited moderate antiproliferative activity toward the cell viability of HeLa, MCF-7, RD, SKOV-3, and SAOS-2 cells with IC_50_ > 15 μg mL^−1^, it showed considerable cytotoxic activity toward PC-3, A-549, CACO2 and MDA-MB-231 cells with IC_50_ > 10 μg mL^−1^. Furthermore, the extract revealed significant antiproliferative activity towards HCT-116, A-431, and HEp-2 cells with IC_50_ < 10 μg mL^−1^. Interestingly, among the cancer cell lines examined, the CIR extract demonstrated the most pronounced antiproliferative activity in cellular viability of HepG-2 and Panc-1 cells with IC_50_ < 5 μg mL^−1^ (IC_50_ = 2.85, and 3.86 μg mL^−1^, respectively). These findings suggest that the extract has promise as a potential antiproliferative with a broad spectrum of antiproliferative activity, particularly for HepG-2 and Panc-1 cells. To examine the effect of the CIR extract on normal cells, we further explored the cytotoxic effect of the extract on normal human fibroblast cells (MRC-5 cell line). In this regard, cells were treated with extract in dose-dependent (0.25–500 μg mL^−1^) and viability was assessed as previously using the MTT assay. As shown in Fig. 3, the extract showed a low antiproliferative activity towards MRC-5 cells with an IC_50_ of 48.70 μg mL^−1^. These findings indicate that the extract has selectivity toward cancer cells with low effect on normal cells, suggesting its antitumor potential. Furthermore, we assessed the selectivity index (SI) of the CIR extract towards the cancer cell lines examined compared to that of the MRC-5 cells. A notable variation was reported among the cancer cell lines tested, with an SI range of 1.7–17.1. The CIR extract showed a moderate selectivity index (SI < 5) toward MCF-7, PC-3, RD, A-549, CACO2, MDA-MB-231, SKOV-3, SAOS-2 and HeLa cells, while it exhibited considerable selectivity (SI > 5) toward HCT-116, A-431, HepG-2, Panc-1and Hep-2 cells, signifying the effect of disparity of the CIR extract on the investigated cell lines. Algin with the observed antiproliferative activity, the CIR extract displayed the highest selectivity index (SI > 10) toward the HepG-2 and Panc-1 cells, indicating the significant selectivity of the CIR extract in those cancer cells. Various studies have explored the phytochemical composition of *C. intybus* and its therapeutical efficacy.^29,108–111^ 3-*O-p*-Coumaroyl quinic acid derived from the *C. intybus* flower extract exhibited antiproliferative activity against PC-3 and undifferentiated noncancerous 3T3L1 fibroblast cells. Furthermore, 5-caffeoylquinic acid substantially inhibited the invasion of nonsmall cell lung cancer cells (H1299).^112^ The *C. intybus* leaf extract showed effective cytotoxicity against LNCaP prostate cancer cells, while the root extract showed significant effectiveness against MCF-7 breast cancer cells, C32 amelanotic melanoma cells and ACHN renal adenocarcinoma cells.^113^ Furthermore, the methanolic extract of *C. intybus* exhibited a time-dependent reduction in the viability of SKBR3 breast cancer cells, with IC_50_ values of 800 μg mL^−1^ at 24 h. In another study, the methanolic extract of *C. intybus* efficiently inhibited the growth of Jurkat cells (IC50 = 138 μg mL^−1^) and moderately inhibited the growth of Fen bladder carcinoma cells and HeLa cervical epithelioid carcinoma cells (25% inhibition at 200 μg mL^−1^).^114^ Aligned with these facts, our study revealed that the CIR extract exerts a noteworthy inhibitory impact on the viability of specific cancer cells, specifically Hep-G2 and Panc-1. Based on the metabolomic analysis of the extract, the pronounced antiproliferative activity of the extract could be attributed to its unique bioactive content, including γ-linolenic acid which has been documented to produce significant antiproliferative and growth inhibitory effects in the treatment of breast and pancreatic cancer cell lines.^115,116^ Furthermore, the presence of acacetin-7-*O*-rutinoside, 3,4,5,7-tetrahydroxyflavanone, which has the potential to serve as anticancer agents with significant antiproliferative capabilities against cancer cells, augments the cytotoxic effect of the extract.^117–119^ Furthermore, the extract includes abundant amounts of gluconate that has well-characterized cytotoxic effects both *in vitro* and *in vivo*.^120^ Moreover, Murugesan *et al.* observed the cytotoxic impact of quinic acid derivatives on human glioblastoma cell lines SNB19 and LN229.^121^ Consequently, the list of bioactive metabolites detected in the CIR extract (Table 1) aligns with the observed antiproliferative potential of the CIR extract against various types of cancer cells.

Based on these results, we explored the morphology of HepG-2, Panc-1, and HCT-116 cells after extract treatment (Fig. 3). The extract was applied at different concentrations (1, 4, 20, 100, and 500 μg mL^−1^) to the cells and the cellular morphology was assessed after a duration of 24 hours under an inverted microscope. As presented in Fig. S1,† the treatment of cells with CIR extract revealed a remarkable pattern of cell loss. Hep-G2, Panc-1, and HCT116 cells, which are polyhedral in shape and typically grow in a tightly connected manner, exhibit normal healthy morphology with a distinct cytoskeleton in the untreated control state. Upon treatment with the CIR extract, the cellular morphology was significantly distorted in a dose-dependent manner. At 4 μg mL^−1^, some cells exhibited a round shape, and areas of cell loss became apparent. With increasing doses, a significant cytotoxic effect was observed, evident in detached cells with characteristic membrane blebbing at 20 μg mL^−1^. In the 100–500 μg mL^−1^ range, cell destruction became more pronounced, with only a few remaining cells attached to the culture dish. These cells displayed distinguishable morphological signs of apoptosis, showing less homogeneity and loss of membrane integrity (Fig. S1†). Our findings showed that the CIR extract has a pronounced cytotoxic effect on HepG-2 and Panc-1 cells. Accordingly, our investigations were directed to explore the underlying mode of action of the observed antiproliferative activity of the extract.

### 3.3. Assessment of cell cycle arrest

**Fig. 4:**
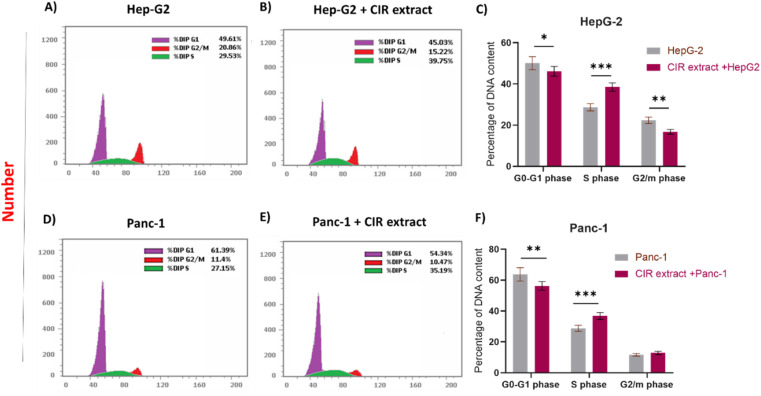
Effects of CIR extract treatment on the distribution of the cell cycle phase. (A) HepG-2 cells without treatment were analyzed by DNA flow cytometry. (B) HepG-2 cells treated with the CIR were analyzed by DNA flow cytometry. (C) Percentage of DNA content compared between treated and untreated HepG-2 cells as distributed in the G0/G1, S and G2/M phases. (D) Panc-1 cells without treatment were analyzed by DNA flow cytometry. (E) Panc-1 cells treated with the CIR and analyzed by DNA flow cytometry. (F) Percentage of DNA content compared between treated and untreated Panc-1 cells as distributed in the G0/G1, S and G2/M phases. Data were expressed as mean ± S. D. in triplicate. Comparisons were made using Student's *t*-tests. Differences between groups were considered significant when *p* < 0.05 (*p < 0.05, **p < 0.01, ***p < 0.001, and ****p < 0.0001).

The MTT analysis demonstrated the substantial ability of the CIR extract to significantly reduce the viability of HepG2 and Panc-1 cells. Consequently, we extended our investigation to explore the impact of CIR extract on the cell cycle of HepG-2 and Panc-1 cells (Fig. 4). To assess this, we conducted a flow cytometry analysis to examine the cell cycle phases in the examined cells after administration of the CIR extract at its IC_50_ value. In the case of HepG-2 cells, treatment with CIR extract (2.85 μg mL^−1^) resulted in a significant decrease in cell percentage in the G1 phase (approximately 5%), and a similar decrease in the G2/M phase (approximately 5.5%), compared to control cells. In contrast, the percentage of the S phase in the extract-treated group was elevated by approximately 10%, indicating blockage of the cell cycle in the S phase (Fig. 4 ACC). In the context of Panc-1 cells, the application of the CIR extract (3.86 μg mL^−1^) resulted in a reduction of approximately 7% in cell percentage in phase G1, compared to control cells. Meanwhile, the phase G2/M exhibited a minor decrease, with a decline from 11.4% to 10.47%. On the other hand, the percentage of cells in phase S increased by around 8% in cells treated with CIR, indicating blockage of the cell cycle in phase S (Fig. 4D–F). These findings indicate that the antiproliferative activity of CIR extract could be associated with its ability to induce cell growth arrest in the S phase. Cell cycle dysregulation is the hallmark of cancer development.^122^ Most human cancers have been reported to originate from disturbances in G1/S cell cycle control.^123,124^ The G1/S phase transition is carefully regulated by various checkpoints. Disruptions in any of the cell cycle regulators or checkpoints, particularly those that guard the transition of cells from the G1 to the S phase, can be debilitating.^124^ Such disturbances may account for uncontrolled proliferation, contributing to the initiation and promotion of cancer; alternatively, they may induce cell death in therapeutic strategies targeting these processes.^122^

The use of drugs or bioactive compounds for the therapeutic manipulation of cell growth arrest in the G1–S phase has demonstrated potent antiproliferative activity and apoptotic effects in many human cancer cell lines. Preclinical studies suggest that cells with impaired checkpoint function are more susceptible to anticancer agents.^122^ Therefore, our findings highlight the impact of CIR extract as an antitumor agent, given its potential to impact crucial cell cycle checkpoints. These findings could be attributed to the presence of specific active metabolites within the CIR extract, such as γ-linolenic acid, acacetin-7-*O*-rutinoside, harmaline and trigonelline. These metabolites have previously been documented for their ability to halt the cell cycle of tumor cells.^125–127^ For example, the Jia-Ren Liu group has documented that linoleic acid can block the cell cycle of the gastric adenocarcinoma cell line SGC-7901.^128^ Furthermore, Liu *et al.* reported that harmaline has the ability to induce cell cycle arrest and trigger cellular apoptosis through the mitochondrial pathway in SW620 cells, through inhibition of the Akt and ERK signaling pathways.^129^ In this regard, this study demonstrated a substantial increase in the population of cells arrested in the S phase, as compared to the control cells. With increasing concentrations of harmaline, the population of cells in the S phase increased in a dose-dependent manner, while the population of cells in the G1 phase decreased. These data are consistent with our findings, underscoring that the cell cycle distribution was significantly arrested in the S phase.^129^ Consequently, it can be inferred that the CIR extract effectively hinders the progression of HepG2 and Panc-1 cells by arresting the cell cycle, thus impeding their replication and proliferation.

### 3.4. Evaluation of programmed cell death (apoptosis and necrosis)

**Fig. 5:**
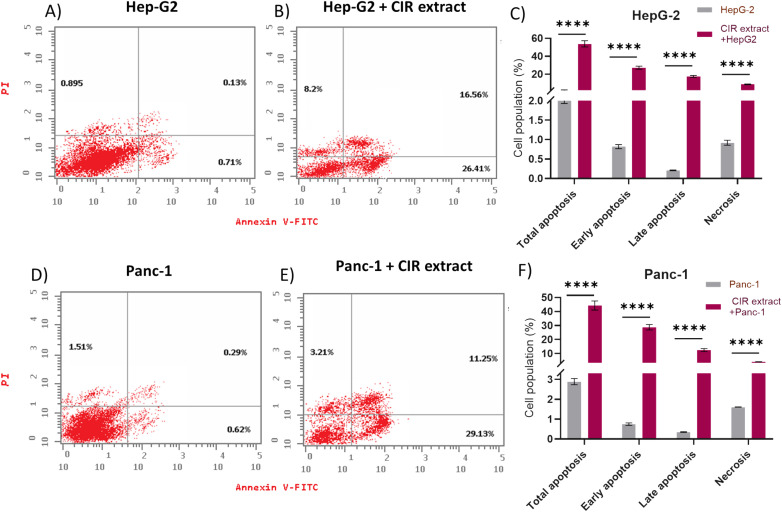
Discrimination of viable cells from cells undergoing apoptosis or necrosis. Hep-G2 cells (untreated) (A) were treated with CIR (B) and then stained with PI and/or Annexin V-FITC. The numbers indicate the percentages of cells in each respective quadrant. (C) Data were calculated for early, late and total apoptosis or necrosis in HepG-2 cells treated or untreated with the extract. Panc-1 cells (untreated) (D) were treated with CIR (E) and then stained with PI and/or Annexin V-FITC. The numbers indicate the percentages of cells in each respective quadrant. (F) Data for early, late and total apoptosis or necrosis were calculated in Panc-1 cells treated or untreated with the extract. Data were expressed as mean ± S. D. in triplicate. Comparisons were made using Student's *t*-tests. Differences between groups were considered significant when *p* ≤ 0.05 (*p ≤ 0.05, **p ≤ 0.01, ***p ≤ 0.001, and ****p < 0.0001).

Next, our objective was to explore whether the antiproliferative effect of the CIR extract is associated with its ability to induce programmed cell death in HepG2 and Panc-1 cells. In this regard, a flow cytometry assay was conducted to analyze the effect of CIR extract on apoptosis and necrosis induction. Cell staining with Annexin V-FITC and PI was used to distinguish and quantitatively determine the percentage of apoptotic and necrotic cells. Viable cells are seen in the left lower quadrant (FITC-/PI−), necrotic cells are seen in the left upper quadrant (FITC-/PI+), early apoptotic cells in the right lower quadrant (FITC+/PI−) and late apoptotic cells in the right upper quadrant (FITC+/PI+).^130,131^ As illustrated in Fig. 5, treatment of HepG-2 cells with CIR extract at the dose of IC50 (2.85 μg mL^−1^) demonstrated the induction of apoptosis in cells, as indicated by the significant increase in total apoptosis in cells treated with CIR. The percentage of early apoptotic cells stained by Annexin V-FITC was approximately 26%, a 37-fold increase compared to control cells, and the percentage of late apoptotic cells double stained by Annexin V-FITC and PI was around 16%, a 127-fold increase compared to control cells. Consequently, total apoptosis increased from 1.73% for control cells to 51.17% for CIR-treated cells, with an almost 30-fold increase. Similarly, necrosis levels showed an increase from 0.89% in untreated control cells to 8.2% in CIR extract-treated cells, demonstrating a 9-fold increase in regulation (Fig. 5C). According to Panc-1 cells, the proportion of early apoptotic cells increased from around 0.6% in control cells to 29% in CIR treated cells (48 times greater), while the percentage of late apoptotic cells increased from 0.29% in control cells to 11.25% in CIR treated cells (39 times greater). Therefore, total apoptotic levels in cells treated with CIR extract reached 43.59% compared to 2.42% in untreated control cells, demonstrating an increase of 18 times. Additionally, necrosis proportions exhibited an approximately twofold upregulation of 1.51% to 3.21% after treatment with CIR extract (Fig. 5D–F). Taken together, these findings indicate that the antiproliferative activity of CIR extract is associated with its ability to substantially trigger programmed cell death pathways, such as apoptosis and necrosis, in cancer cells and further underscore the pharmacological potential of CIR extract as a natural-based antitumor supplement.

### 3.5. Evaluation of apoptosis-related gene expression

**Fig. 6:**
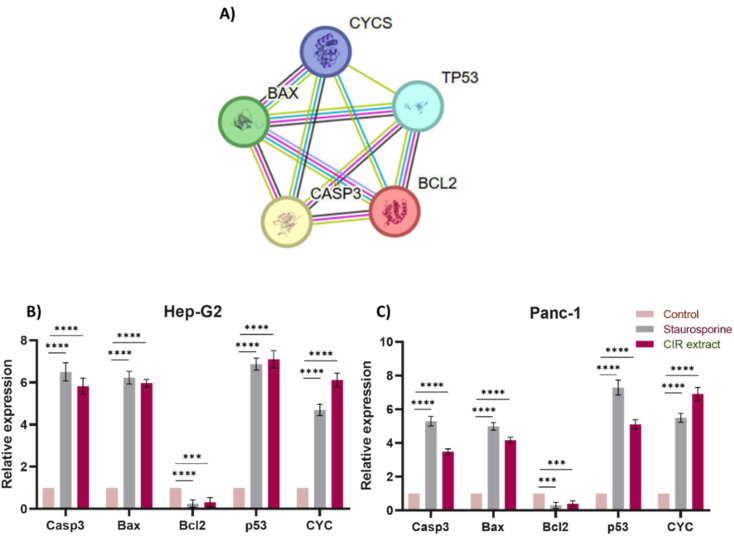
Effect of the CIR extract on the expression of apoptotic genes in Hep-G2 and Panc-1 cells. (A) Protein–protein interaction network for the investigated genes involved in apoptosis. The nodes represent proteins, and the edges denote predicted associations. The colored lines indicate different types of evidence, with red representing fusion evidence, light blue representing database evidence, green representing neighborhood evidence, blue representing co-occurrence evidence, purple representing experimental evidence, black representing co-expression evidence and yellow representing text mining evidence, interaction scores are detailed in supplementary Table S5.† Relative gene expression of the Casp3, Bax, Bcl-2, P53, and CYC genes in Hep-G2 (B) and Panc-1 (C) cells after treatment with CIR extract (at IC_50_ value) or staurosporine, compared to control cells. Data were expressed as mean ± S. D. in triplicate. Comparisons were made using the ANOVA test and Tukey as a *post hoc* test. Differences between groups were considered significant when *p* ≤ 0.05 (*p ≤ 0.05, **p ≤ 0.01, ***p ≤ 0.001, and ****p < 0.0001).

To gain further insight into the apoptotic mode of action of the CIR extract, we extended our investigation to assess the expression of key apoptotic-associated genes in HepG2 and Panc-1 cells. The intrinsic apoptotic pathway is regulated by the Bcl-2 (B cell lymphoma 2) family of proteins. The proteins in this family can be classified by their anti-apoptotic (such as Bcl-2) or pro-apoptotic (such as Bax) actions. Activation of these pathways results in activation of caspases that lead to apoptosis.^132^ In particular, the pro-apoptotic gene Bax is one of the downstream effectors of p53, which is a main tumor suppressor in multicellular organisms that induces cell cycle arrest, DNA repair, and apoptosis to promote genomic stability and tissue homeostasis.^133^ A variety of apoptotic stimuli cause mitochondrial cytochrome c release, which in turn induces a series of biochemical reactions that result in caspase activation and subsequent cell death.^134^ The crosstalk between these genes and their role in inducing and controlling apoptosis is demonstrated in the apoptotic pathway and protein interactions, as indicated by the KEGG and String databases (Fig. 6 and S2†).^46,135^ Toward this end, we evaluated the expressions of the Casp3, Bax, Bcl-2, P53 and CYC genes after cells were treated with CIR extract at the corresponding IC_50_ values.

In our investigations, staurosporine, a recognized apoptotic inducer, was used as a control reference drug. As shown in Fig. 6, treatment of HepG2 cells with CIR extract at 2.85 μg mL^−1^ demonstrated the ability to significantly upregulate the expression of Casp3 (5.8 times), Bax (5.9 times), P53 (7 times) and CYC genes (6 times), compared to control HepG2 cells. However, it considerably decreased the expression of the Bcl2 gene (0.3-fold change), compared to untreated cells. Interestingly, the efficacy of CIR extract was comparable to that of the well-known staurosporine drug which showed the ability to increase the expression of Casp3 (6.5 times change), Bax (6.2 times change), P53 (6.9 times change) and CYC genes (4.7 times change), while it reduced the expression of the Bcl2 gene (0.2 times change). Regarding Panc-1 cells, treatment with CIR extract at 3.86 μg mL^−1^ demonstrated the ability to substantially increase the expression of the Casp3 (3.4-fold change), Bax (4.2-fold change), P53 (5-fold change) and CYC genes (6.8-fold change), compared to control Panc-1 cells. However, it considerably mitigated the expression of the Bcl2 gene (0.38-fold change), compared to untreated cells. Similarly, comparing the efficacy of CIR extract with that of the well-known staurosporine drug showed the ability to increase the expression of the Casp3 (5.2 times), Bax (4.9 times), P53 (7.3 times) and CYC genes (5.5 times), while reducing the expression of the Bcl2 gene (0.29 times) (Fig. 6). These results further indicate that the antiproliferative activity of CIR extract could be related to its potential ability to modulate the expression of apoptotic genes in cancer cells. The observed apoptotic effect of the CIR extract can be attributed to the presence of several bioactive metabolites, including γ-linolenic acid,^136,137^ 3′,4′,5,7-tetrahydroxyflavanone,^138^*trans*-cinnamate^139^ and naringenin.^140,141^ Mechanistically, γ-linolenic acid showed the potential to induce apoptosis in K562/ADM cells by triggering lipid peroxidation and caspase-3.^137^ The necrotic effect of γ-linolenic acid was also demonstrated in human gliomas where it induced extensive necrosis and cystic degeneration.^142^ Furthermore, naringenin showed a strong antitumor effect in MDA-MB-231 cells by blocking cells in the G0/G1 phase, altering the mitochondrial-mediated intrinsic pathway responsible for apoptosis and inducing inflammatory reaction.^141^ Furthermore, the apoptotic effect of naringenin was related to its ability to modulate pro-apoptotic genes including the Bax, P53 and Casp3 genes.^143,144^ Kanno *et al.* 2006 showed that naringenin induces necrotic activity in human pro-myeloleukemia HL-60 cells and triggers mitochondrial dysfunctions by depleting intracellular ATP.^145^ Collectively, the remarkable cytotoxic activity of CIR extract in the cellular growth of cancer cells could be further explained by the unique phytochemical profile of CIR extract, which possesses a set of bioactive metabolites that significantly trigger apoptotic levels in cancer cells. These findings further affirm the antitumor potential of the CIR extract and support CIR as a natural pharmacological supplement for human diseases.

### 3.6. Evaluation of anti-inflammatory activity

To further explore the pharmacological impacts of CIR extract, we aimed to explore its anti-inflammatory activity by assessing its ability to target the pro-inflammatory enzymes COX-1 & 2 and to mediate the expression of inflammation-related genes (IL-1b, IL-6, and TNF-α genes).

#### 3.6.1. Evaluation of cyclooxygenases activity

**Fig. 7:**
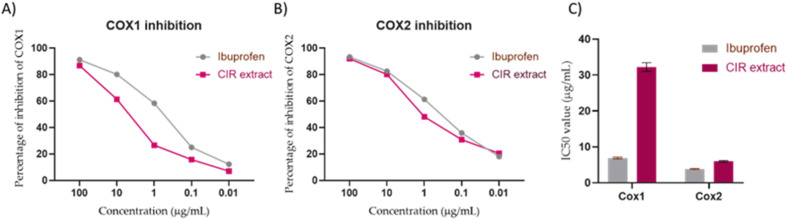
The inhibitory activity of CIR extract on the activity of COX1 (A) and COX2 (B), compared to the ibuprofen drug. (C) Representation of the IC_50_ values of CIR extract and ibuprofen toward the activity of the COX1 and COX2 enzymes. Data were expressed as mean ± S. D. in triplicate.

Cyclooxygenases play a crucial role in the conversion of arachidonic acid to prostaglandins that are involved in inflammatory conditions, including cancer.^146^ To further explore the therapeutic impact of CIR extract, we evaluated the inhibitory activity of CIR extract on the enzymes of cyclooxygenases (COX-1 and COX-2) enzymes.^147^ In this regard, ibuprofen was served as the control reference drug. As shown in Fig. 7, the CIR extract showed a substantial and dose-dependent inhibitory activity toward COX-1 activity. At a concentration of 100 μg mL^−1^ concentration, the CIR extract exhibited 87% inhibitory activity, compared to ibuprofen (91.3%). At lower concentrations (0.01–10 μg mL^−1^), the extract had inhibitory activity of 7.1–61.4%, compared to ibuprofen (12.3–80%). Although our analysis indicated that the CIR extract exhibits considerable inhibitory activity of COX-1, it displayed an IC_50_ of 32.24 μg mL^−1^ with 4.6 times less activity than ibuprofen (IC_50_ = 6.9 μg mL^−1^). Regarding the evaluation of COX-2 activity, the CIR extract showed a pattern similar to that of COX-1, with significant concentration-dependent enzyme inhibition. The inhibitory activity of CIR extract ranged from 20.5–92% at concentrations of 0.01–100 μg mL^−1^, as compared to that of ibuprofen (18–93%). Interestingly, our findings revealed that CIR extract has inhibitory activity towards the COX2 enzyme (IC_50_ = 5.97 μg mL^−1^) similar to that of the reference ibuprofen drug (IC_50_ = 3.8 μg mL^−1^). Although the CIR extract demonstrated a considerable inhibitory effect toward the COX-1 and COX-2 enzymes, our findings revealed that the CIR possesses a pronounced selectivity toward the activity of COX-2, with a potency comparable to ibuprofen (Fig. 7). The observed selectivity toward COX enzymes could be attributed to γ-linolenic acid, the most prominent metabolite detected in the CIR extract, which has been recognized for its selective activity toward COX-2 enzyme.^148^ Taken together, our findings indicate that CIR extract exhibits considerable anti-inflammatory potential and further reveal that the remarkable antiproliferative activity of CIR extract could be associated with the ability of CIR extract to substantially target the COX-2 enzyme.

#### 3.6.2. Evaluation of inflammation-related gene expression

**Fig. 8:**
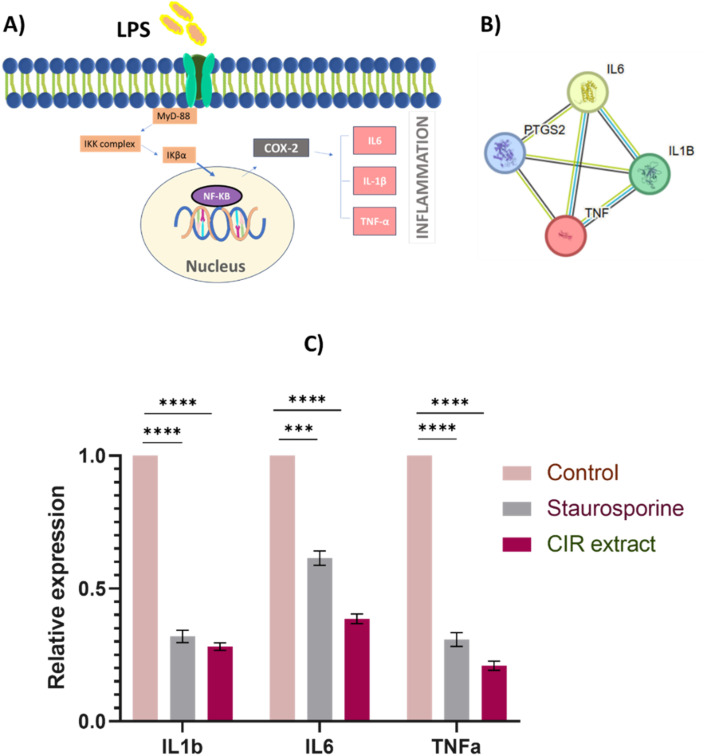
Effect of the CIR extract on the expression of inflammatory genes in LPS-induced RAW 264.7 cells. (A) The proposed KEGG pathway shows IL-1b, IL-6 and TNF-α as selected inflammatory markers associated with the COX2 mediator. (B) Protein–protein interaction network for the investigated genes involved in the inflammatory process. The nodes represent proteins, and edges denote predicted associations. The colored lines indicate different types of evidence, with red representing fusion evidence, light blue representing database evidence, green representing neighborhood evidence, blue indicating co-occurrence evidence, purple representing experimental evidence, black indicating co-expression evidence, and yellow representing text mining evidence, interaction scores are detailed in supplementary Table S6.† STRING analysis^135^ (C) the relative expression of the genes IL-1b, IL-6 and TNF-α genes in LPS-induced RAW 264.7 cells after treatment with CIR extract (at IC_50_ value) or staurosporine, compared to control cells. Data were expressed as mean ± S. D. in triplicate. Comparisons were performed using the ANOVA test and Tukey as a *post hoc* test. Differences between groups were considered significant when *p* ≤ 0.05 (*p ≤ 0.05, **p ≤ 0.01, ***p ≤ 0.001, and ****p < 0.0001).

To further investigate the anti-inflammatory activity of the CIR extract, we envisaged evaluating the expression of inflammatory-related genes in LPS-induced RAW 264.7 cells.^149^ Toward this, we performed string analysis and checked the KEGG pathway database which revealed that the expression of the IL-1b, IL-6 and TNF-α gene is associated with the COX2 mediator (Fig. 8 and S3†).^46,150^ Consequently, we evaluated the expression of the IL-1b, IL-6, and TNF-α genes in LPS-induced RAW 264.7 cells after CIR extract treatment. The anti-inflammatory activity of the CIR extract was compared to that of the anticancer staurosporine drug.^151^ As shown in Fig. 8, CIR extract treated cells exhibited a significant down-regulation in the expression of the IL-1b (0.2783-fold change), IL-6 (0.3803-fold change) and TNF-α genes (0.21-fold change), compared to untreated cells. Similarly, staurosporine-treated cells showed substantial mitigation in the expression of IL-1b (0.3204 times change), IL-6 (0.6129 times change) and TNF-α genes (0.3104 times change), compared to control cells (Fig. 8). These findings indicate that the CIR extract has potential anti-inflammatory activity compared to that of the staurosporine drug. Interestingly, CIR extract exhibited more potency (one-fold) to down-regulate the expression of the pro-inflammatory IL-6 gene, compared to staurosporine. These findings further emphasize the remarkable potency and selectivity of CIR extract with respect to the COX enzymes. Our results coincide with studies by Silva *et al.* and Conforti *et al.* studies^152,153^ which reported the anti-inflammatory effects of botanical leaf extracts of evergreen shrubs growing in South America and Mediterranean dietary plants. The notable potency of the CIR extract could be associated with the detected phytochemical metabolites with considerable anti-inflammatory activity, including γ-linolenic acid, acacetin-7-*O*-rutinoside, 3′,4′,5,7-tetrahydroxyflavanone, naringenin, *trans*-cinnamate, harmaline, and *N*,*N*-dimethylglycine.^154–158^ For example, harmine exhibited the ability to inhibit TNF-α, NF-κB transitivity, and inflammation in LPS-induced RAW 264.7 cells.^155^ Further, γ-linolenic acid and harmine showed a considerable ability to modulate immune and inflammatory activities by mitigating the expression of TNF-α, IL-1b, IL-2, and IL-6 genes.^155,159^ Tumors and tumor microenvironments are infiltrated by a diverse group of immune and non-immune cells whose functions can have both pro- and anti-tumor effects. The balance of these opposing inflammatory mediators plays a pivotal role in determining tumor progression and treatment outcome.^160^ Additionally, chronic inflammation increases the risk of various cancers, indicating that targeting inflammation may represent a valid strategy for cancer prevention and therapy. Furthermore, cancer-related inflammation is characterized by the presence of inflammatory cells and the up-regulation of inflammatory mediators such as cytokines, and prostaglandins.^161^ In addition, persistent inflammation has been recognized as an established factor that increases the risk of developing several types of cancer.^161^ The implications of using an anti-inflammatory agent to treat cancers and modulate their cytotoxicity has been well documented.^162,163^ In this regard, the present findings indicate that the CIR extract exhibits pronounced selectivity toward COX-2 activity, while also significantly negatively regulating the expression of IL-1β, IL-6, and TNF-α genes. This further emphasizes the pharmacological impact of CIR extract as a potent natural anti-inflammatory and antitumor supplement.

### 3.7. Assessment of antioxidant activity

**Fig. 9:**
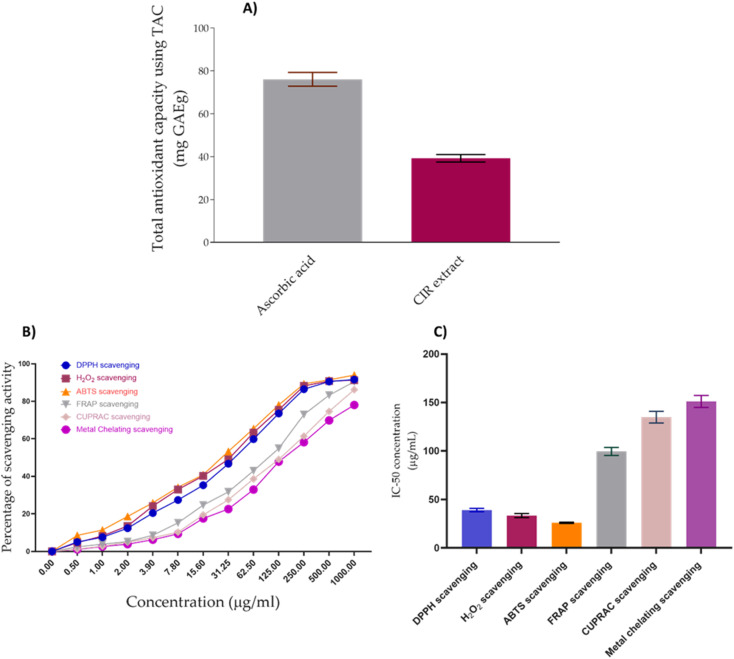
The antioxidant activity of the CIR extract. (A) The total antioxidant capacity of the CIR extract, as compared to ascorbic acid. (B) The dose-dependent scavenging activity of the CIR extract toward the DPPH radical, the H_2_O_2_ radical, the ABTS radical, FRAP, CURPAC, and metal chelating activity. (C) Representative diagram for the IC_50_ values of CIR extract in relation to DPPH, H_2_O_2_, ABTS, FRAP, CURPAC, and metal chelating scavenging assays. Data were expressed as mean ± S. D. in triplicate.

Cancer has been linked to ROS-mediated damage to biological macromolecules, which arises from an imbalance between radical generating and radical-scavenging systems.^164^ In this regard, we have extended our investigations to explore the antioxidant impact of CIR extract. To achieve this aim, we have initially assessed the total antioxidant capacity (TAC) of the CIR extract. Our analysis revealed that the CIR extract has considerable antioxidant capacity with a TAC of 39.21 ± 1.75 mg GAE per g, compared to ascorbic acid (TAC = 73.6 mg GAE per g). Based on these results, we extended our explorations to assess the antioxidant activity of CIR extract by examining its free radical scavenging activity against DPPH, ABTS and H_2_O_2_ radicals, but also its metal scavenging activity (FRAP, metal chelating and CUPRAC scavenging assays). In our investigations, ascorbic acid and EDTA functioned as the standard positive control (ESI Fig. S4–S6†). Evaluation of the free radical scavenging activity of CIR extract towards DPPH, ABTS, and H_2_O_2_ radicals revealed that it has substantial and dose-dependent scavenging activity, compared to ascorbic acid (Fig. 9).

IC_50_ evaluation indicated that CIR exhibits an inhibitory activity of 39.06 ± 1.78 μg mL^−1^ toward DPPH radicals, 26.03 ± 0.69 μg mL^−1^ toward the ABTS radicals, and 33.36 ± 2.09 μg mL^−1^ toward the H_2_O_2_ radicals, compared to ascorbic acid (IC_50_ = 10.19 ± 0.75 μg mL^−1^, 10.66 ± 0.89, 14.77 ± 0.69, respectively). These results indicate that the CIR extract exhibits substantial antioxidant activity by scavenging free radicals. Similarly, an evaluation of the metal scavenging activity of the CIR extract using FRAP, metal chelating, and CUPRAC scavenging assays indicated that CIR extract displays considerable antioxidant activity in a concentration-dependent manner (Fig. 9). Our analysis revealed that CIR shows a considerable reducing potential toward copper (Cu^2+^) and iron (Fe^3+^) ions, as indicated by CUPRAC and FRAP assays, with an IC_50_ of 134.82 ± 5.98, and 99.50 ± 4.09 μg mL^−1^, respectively. Furthermore, the CIR extract exhibited satisfactory metal chelating activity with an IC_50_ of 151.1 ± 6.14 μg mL^−1^. These findings indicate that the CIR extract possesses significant reduction power and metal chelating activity, further highlighting the antioxidant potential of the CIR extract. The observed antioxidant capacity of CIR extract could be correlated with the unique content of bioactive metabolites present in the extract, as described in Table 1. Several detected metabolites present with a high prevalence in the CIR extract, including γ-linolenic acid, acacetin-7-*O*-rutinoside, 3′,4′,5,7-tetrahydroxyflavanone, naringenin, *N*,*N*-dimethylglycine, apigenin, and gluconate, are well recognized for their significant antioxidant capacity.^165–167^ In alignment with our findings, Celep *et al.* showed that a three-edible fruit extract exhibits a substantial antioxidant capacity, as evidenced by the observed potent free radical and metal scavenging activities.^168^ Similar to the CIR extract presented, the phytochemical profile of the investigated extract showed an abundance of flavonoids, including acacetin-7-*O*-rutinoside, naringenin, and apigenin.^168^

### 3.8. Molecular modeling study

To gain more insight into the anti-inflammatory activity of the CIR extract and its selectivity to cyclooxygenase enzymes (COX-1 & 2), we extended our investigation to explore the binding affinity of the most prominent secondary metabolites detected in the CIR extract to the binding cavity of the COX-1 and COX-2 proteins. In this regard, we conducted extensive molecular coupling studies for a set of 12 secondary metabolites, including acacetin-7-*O*-rutinoside; 3′,4′,5,7-tetrahydroxy-flavanone; naringenin; 3,5,7-trihydroxy-4′-methoxy-flavone; harmaline; *trans*-cinnamate; γ-linolenic acid; gluconate; isorhamnetin-3-*O*-glucoside; 12-oxo-10,15-(*Z*)-phytodienoic acid; apigenin and peonidine-3-*O*-glucoside. The crystal structures of COX-1 & 2 were selected and acquired from PDB, and the applied protocol was adjusted to confirm the essential interactions reported for the co-crystallized ligand.

#### 3.8.1. Cyclooxygenase 1 (COX-1)

**Fig. 10:**
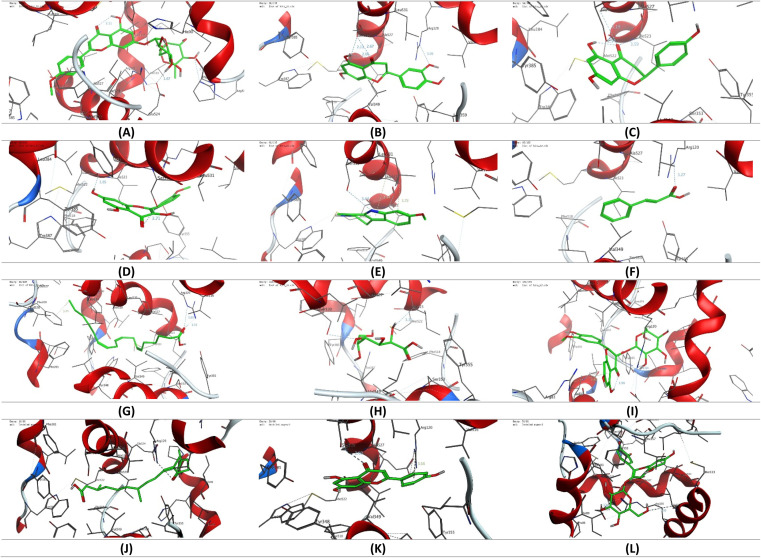
3D representation of the binding mode of selected secondary metabolites identified in the CIR extract to the binding cavity of the COX-1 protein (PDB code: 1eqg). (A) Acacetin-7-*O*-rutinoside; (B) 3′,4′,5,7-tetrahydroxy-flavanone; (C) naringenin; (D) 3,5,7-trihydroxy-4′-methoxy-flavone; (E) harmaline; (F) *trans*-cinnamate; (G) γ-linolenic acid; (H) gluconate; (I) isorhamnetin-3-*O*-glucoside; (J) 12-oxo-10,15(*Z*)-phytodienoic acid; (K) apigenin; (L) peonidine-3-*O*-glucoside.

Toward the COX-1 protein, a molecular coupling study was performed using the reported crystal structure (PDB code: 1eqg) with ibuprofen as a cocrystallized ligand. Aligned with the reported data, our analysis revealed that ibuprofen, through its carboxyl group, possesses two essential H-binding interactions with the residues Arg120 and Tyr355, along with a set of hydrophilic interactions (Table 2). Consequently, the adjusted protocol was applied to explore the affinity of the selected metabolites. As shown in Fig. 10, the examined metabolites exhibited thermodynamically favorable binding with considerable affinity scores toward the active site of the COX-1 enzyme (Table 2). Among the metabolites examined, only the isorhamnetin-3-*O*-glucoside showed the ability through its glucoside moiety to interact with the essential amino acids residues Arg120 and Tyr355. Furthermore, the isorhamnetin-3-*O*-glucoside formed an additional H-arene interaction with the Ile89 residue. On the other hand, acacetin-7-*O*-rutinoside, 3′,4′,5,7-tetrahydroxy-flavanone, γ-linolenic acid, apigenin, and peonidine-3-*O*-glucoside demonstrated the ability to interact with only one essential amino acid residue (Arg120), together with the ability to bind to other amino acid residues. In this regard, acacetin-7-*O*-rutinoside and peonidine-3-*O*-glucoside exhibited the ability through the sugar residue to form two additional hydrogen interactions with the residues Glu524, and Arg83 and Glu524, Val116 residues, respectively. While γ-linolenic acid, 3′,4′,5,7-tetrahydroxy-flavanone, and apigenin showed the ability to form only one extra-hydrogen binding with the residues Phe209 and Ser530, respectively. Although the metabolites of *trans*-cinnamate and 12-oxo-10,15-(*Z*)-phytodienoic acid displayed only one hydrophilic interaction with the Arg120 residue *via* the carboxylic moiety, their hydrophobic nature supported their affinity for the COX-1 cavity by forming a set of hydrophobic interactions with various residues. The other metabolites including 3,5,7-trihydroxy-4′-methoxy-flavone, harmaline, and glucose revealed a different binding mode other than the original ligand. In this binding mode, the Ser530 residue played a critical role in the binding of the examined metabolite (Fig. 10 and S7†). These metabolites showed a considerable interaction with the Ser530 residue together with an additional interaction with the Met522, Ala527, and Ile523 residues, respectively. Finally, naringenin showed a considerable hydrophilic interaction with the Ile523 and Gly526 residues together with a set of hydrophobic interactions. However, the affinity score indicated that naringenin has a low affinity for the COX-1 pocket. Taken together, our findings reveal that the examined metabolites, especially isorhamnetin-3-*O*-glucoside acacetin-7-*O*-rutinoside and peonidine-3-*O*-glucoside, possess considerable affinity for the COX-1 pocket and further support the considerable inhibitory activity observed of CIR extract toward COX-1 activity.

**Table 2:** The list of binding interactions and affinity of selected secondary metabolites identified in CIR extract toward the binding cavity of COX-1 protein (PDB code: 1eqg)

Ligand	Score (kcal mol^−1^)	Interactions	
		Hydrophilic	Hydrophobic
Ibuprofen	−10.26	Arg120 (2.91 Å, 3.59 Å, 2.83 Å), Tyr355 (2.78 Å)	Val116, Leu531, Val349, Leu352, Met522, Ala527, Ile523, Tro387, Phe518, Leu359
Acacetin-7-*O*-rutinoside	−9.35	Arg120 (2.56 Å), Glu524 (2.62 Å), Arg83 (2.78 Å)	Leu93, Met522, Trp387, Leu384, Val116, Leu531, Val349, Leu115, Leu359, Met113, Ala527, Val119, Phe86, Ile89
3′,4′,5,7-Tetrahydroxy-flavanone	−8.62	Ser530 (2.88 Å, 2.67 Å), Arg120 (3.09 Å)	Phe518, Leu359, Val116, Ala527, Trp387, Leu531, Leu352, Ile523, Val349, Met522
Naringenin	−6.18	Ile523 (3.59 Å), Gly526 (3.19 Å)	Met522, Leu384, Phe381, Ala527, Phe518, Leu531, Trp387, Val116, Val344, Val349, Leu352
3,5,7-Trihydroxy-4′-methoxy-flavone	−7.04	Ser530 (2.71 Å), Met522 (2.75 Å)	Leu531, Val349, Val116, Phe518, Ile523, Trp387, Ala527, Leu384, Phe381
Harmaline	−7.61	Ser530 (3.44 Å), Ala527 (3.94 Å, 3.72 Å)	Met522, Leu531, Leu352, Ile523, Leu359, Val116, Val349, Phe518, Trp387
*trans*-Cinnamate	−7.83	Arg120 (3.27 Å)	Leu352, Ile523, Val116, Phe518, Val527, Val349, Leu531
γ-Linolenic acid	−8.96	Arg120 (3.03 Å, 3.13 Å), Phe209 (3.74 Å)	Met522, Phe205, Leu534, Val228, Ile523, Leu531, Ala527, Val116, Val349, Phe381, Trp387, Val344, Leu352
Gluconate	−6.79	Ser530 (2.83 Å), Ile523 (3.36 Å)	Val349, Phe518, Met522, Ala527, Trp387, Leu352
Isorhamnetin-3-*O*-glucoside	−12.17	Ile89 (3.82 Å), Arg120 (2.64 Å), Tyr355 (2.62 Å)	Pro86, Leu93, Leu359, Val349, Ile523, Val116, Leu531, Ala527, Leu357, Ile112, Leu115, Trp100
12-Oxo-10,15(*Z*)-phytodienoic acid	−8.14	Arg120 (3.03 Å)	Leu531, Ile523, Val116, Met522, Ala527, Phe518, Pro86, Ile89, Leu93, Leu352, Val349, Trp387
Apigenin	−8.47	Arg120 (3.98 Å), Ser530 (2.6 Å)	Val116, Phe518, Trp387, Met522, Val349, Ile523, Leu352, Leu531, Leu534, Ala527
Peonidine-3-*O*-glucoside	−9.11	Arg120 (2.73 Å), Glu524 (2.96 Å), Val116 (3.30 Å)	Ile89, Ile521, Leu93, Pro86, Ala527, Met522, Phe518, Leu352, Met113, Ile345, Val349, Leu354, Leu357

#### 3.8.2. Cyclooxygenase 2 (COX-2)

**Fig. 11:**
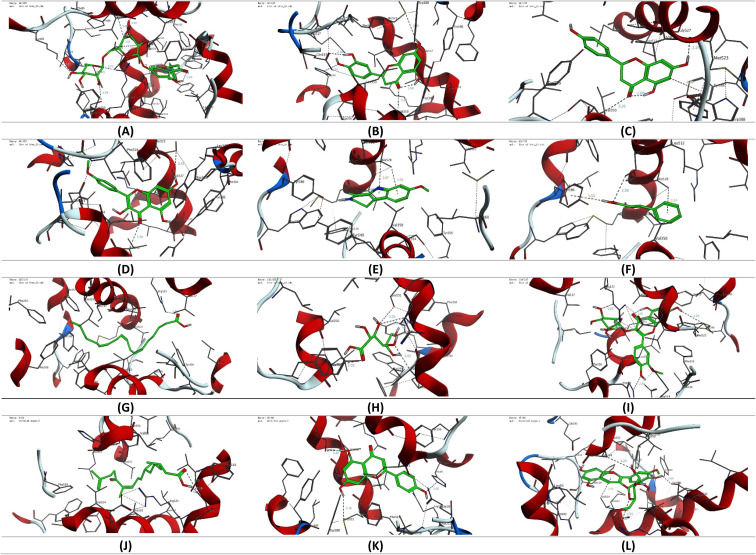
3D representation of the binding mode of selected secondary metabolites identified in the CIR extract toward the binding cavity of the COX-2 protein (PDB code: 4ph9). (A) Acacetin-7-*O*-rutinoside; (B) 3′,4′,5,7-tetrahydroxy-flavanone; (C) naringenin; (D) 3,5,7-trihydroxy-4′-methoxy-flavone; (E) harmaline; (F) *trans*-cinnamate; (G) γ-linolenic acid; (H) gluconate; (I) isorhamnetin-3-*O*-glucoside; (J) 12-oxo-10,15(*Z*)-phytodienoic acid; (K) apigenin; (L) peonidine-3-*O*-glucoside.

Our investigations were carried out using the reported crystal structure of the COX-2 protein (PDB code: 4ph9) with ibuprofen as a ligand. Our results showed that ibuprofen binds through its carboxyl group to the COX-2 cavity by forming three H-binding interactions with the residues Arg121 and Tyr356. Furthermore, the affinity was enhanced by hydrophobic interactions with grassy amino acid residues (Table 3). These findings were in agreement with the crystal structure of the ibuprofen–COX-2 complex. Based on these results, we used our protocol to investigate the binding affinity of the selected secondary metabolites of the CIR extract. As indicated in Table 3, our analysis revealed that the examined metabolites exhibit a substantial binding affinity to the active pocket of the COX-2 enzyme by forming a thermodynamically stable binding to a set of amino acid residues in the enzyme cavity. Among the examined metabolites, acacetin-7-*O*-rutinoside, isorhamnetin-3-*O*-glucoside and peonidine-3-*O*-glucoside demonstrated the highest affinity score for the COX-2 pocket, compared to the original ibuprofen ligand. As shown in Fig. 11, these metabolites were able to bind the essential amino acid residues Arg121 and Tyr356, but also to form additional H binding with other residues. In this sense, the acacetin-7-*O*-rutinoside interacted with 5 additional amino acid residues, including Glu525, Arg514, Pro86, Ser120, and Lys83, to form an H-binding network of 9 hydrophilic interactions. Furthermore, the stability of the ligand–protein complex was further enhanced by the hydrophobic interactions between the flavonoid moiety and the grassy amino acids in the enzyme pocket (14 residues). Similarly, isorhamnetin-3-*O*-glucoside showed additional hydrophilic interactions with Met523 and His90 residues, along with a set of hydrophobic interactions that contributed to the enhancement of the binding affinity to the COX-2 pocket. Although the peonidin-3-*O*-glucoside binding mode showed the essential interaction with only the residue Arg121, it interacted with an additional 3 amino acids, including the residues Ser354, Ser531, and Val524 to provide a hydrophilic network of 5 hydrogen bindings (Fig. 11 and S8†). The affinity of peonidin-3-*O*-glucoside was further extended by hydrophobic bindings to amino acid residues at the active protein site (10 residues). Relatedly, γ-linolenic acid showed only one hydrophilic interaction with the Arg121 residue through its carboxyl moiety. However, the hydrophobic tail contributed to the enhancement of binding affinity by forming a hydrophobic interaction network with 13 amino acid residues in the active pocket of COX-2. The other metabolites examined, including 3′,4′,5,7-tetrahydroxy-flavanone, gluconate, 3,5,7-trihydroxy-4′-methoxy-flavone, apigenin, naringenin, harmaline and *trans*-cinnamate, exhibited a different binding mode than the original ibuprofen ligand. The best binding mode was observed for gluconate and 3′,4′,5,7-tetrahydroxy-flavanone with binding scores of −10.26 and −9.45 kcal mol^−1^, respectively. The gluconate showed a hydrophilic binding mode with 4 amino acid residues, including the Ala528, Val524, Ser353 and Ser531 residues, along with hydrophilic interactions with 7 amino acid residues in the COX-2 pocket. Similarly, 3′,4′,5,7-tetrahydroxyflavanone showed another binding mode with 4 hydrogen binding interactions (Met523, Glu193, His90, and Val350 residues), and hydrophilic interactions with 8 amino acid residues. The considerable binding score of gluconate and 3′,4′,5,7-tetrahydroxyflavanone indicate that the observed binding mode is quite thermodynamically favorable and comparable in affinity to ibuprofen. Furthermore, apigenin and *trans*-cinnamate exhibited a binding mode to the COX-2 pocket with considerable affinity (−8.70 and −8.39 kcal mol^−1^, respectively). The observed affinity scores could be associated with their ability to form H-bound interactions with several amino acid residues. As indicated in Fig. 11, apigenin showed a binding mode with 3H bindings to the Ser531, Met523, and His90 residues, while *trans*-cinnamate showed an interaction with the Try386, Ala528, and Ser531 residues. Our findings indicated that the Ser531 residue could be beneficial for the stability of the ligand–protein complex. Interestingly, our analysis revealed that naringenin and 3,5,7-trihydroxy-4′-methoxy-flavone exhibited similar binding modes and affinities when interacting with Met523 and Val350 residues. On the other hand, harmaline and 12-oxo-10,15(*Z*)-phytodienoic acid showed moderate affinity for the active site of the COX-2 protein *via* hydrophilic interaction with only one amino acid residue. Collectively, molecular coupling studies indicate that the examined metabolites of the CIR extract possess a substantial affinity for the COX-2 protein, which could be associated with the observed selectivity of the CIR extract towards COX-2 rather than COX-1. Additionally, our findings suggest that acacetin-7-*O*-rutinoside, isorhamnetin-3-*O*-glucoside and peonidine-3-*O*-glucoside could be potential antagonists of COX-2 activity, which warrants further exploration of their potency.

**Table 3:** The list of binding interactions and affinity of selected secondary metabolites identified in the CIR extract toward the binding cavity of the COX-2 protein (PDB code: 4ph9)

Ligand	Score (kcal mol^−1^)	Interactions	
		Hydrophilic	Hydrophobic
Ibuprofen	−11.36	Arg121 (3.67 Å, 2.97 Å, 3.01 Å), Tyr356 (2.42 Å)	Leu532, Leu360, Ala528, Phe519, Val117, Met523, Val524, Val350, Trp388, Leu353
Acacetin-7-*O*-rutinoside	−13.51	Arg121 (2.79 Å, 2.61 Å), Tyr356 (2.45 Å), Arg514 (2.93 Å), Glu525 (2.54 Å, 3.03 Å), Pro86 (3.72 Å), Ser120 (2.79 Å), Lys83 (2.83 Å)	Lys360, Val350, Met523, Phe382, Ala528, Val524, Leu385, Leu353, Phe519, Val89, Leu93, Val117, Trp388, Phe471
3′,4′,5,7-Tetrahydroxy-flavanone	−9.45	Met523 (4.25 Å), Glu193 (3.26 Å), His90 (3.03 Å), Val350 (3.27 Å)	Ala528, Leu385, Val524, Trp388, Phe519, Leu353, Ile518, Ala517
Naringenin	−7.88	Met523 (3.26 Å), Val350 (3.26 Å)	Leu532, Leu353, Val524, Trp388, Leu385, Ala528, Val117
3,5,7-Trihydroxy-4′-methoxy-flavone	−7.16	Val350 (3.3 Å), Met523 (4.26 Å)	Try388, Leu353, Ala517, Phe519, Leu385, Val524, Phe382, Ala528
Harmaline	−6.52	Ala528 (3.59 Å, 3.97 Å)	Phe519, Val117, Leu360, Leu532, Val524, Meth523, Leu353, Val350, Trp388
*trans*-Cinnamate	−8.39	Try386 (4.64 Å), Ala528 (3.54 Å), Ser531 (2.98 Å)	Val350, Leu532, Leu353, Val524, Trp388, Leu385
γ-Linolenic acid	−9.11	Arg121 (2.93 Å)	Val345, Trp388, Val350, Phe206, Phe210, Phe382, Val524, Leu353, Ala528, Leu535, Leu532, Val117, Leu93
Gluconate	−10.26	Ala528 (3.44 Å), Val524 (3.43 Å), Ser353 (3.5 Å), Ser531 (3.39 Å)	Trp388, Phe519, Met523, Leu385, Phe382, Leu353, Val350
Isorhamnetin-3-*O*-glucoside	−12.40	Met523 (4.25 Å), Tyr356 (2.5 Å), Arg121 (2.5 Å, 3.25 Å), His90 (2.74 Å)	Phe382, Phe519, Ala517, Ile518, Leu360, Val117, Val350, Leu353, Leu532, Leu385, Val524, Trp388, Ala528
12-Oxo-10,15(*Z*)-phytodienoic acid	−6.94	Ser120 (2.93 Å, 3.23 Å)	Phe519, Val524, Leu532, Leu353, Val350, Leu360, Val89, Leu93, Val117
Apigenin	−8.70	Ser531 (2.83 Å), Met523 (4.09 Å), His90 (2.92 Å)	Val350, Ala528, Trp388, Val524, Leu385, Phe519, Leu353, Ala517, Ile518
Peonidine-3-*O*-glucoside	−11.65	Arg121 (3.54 Å, 2.88 Å), Ser354 (3.28 Å), Ser531 (2.81 Å), Val524 (3.43 Å)	Phe302, Phe519, Trp388, Ala517, Met523, Val350, Leu532, Leu360, Ala528, Val117

## 4 Conclusion

In conclusion, this study bridged the gap in the limited explorations of the pharmacological potential of the CIR extract. The conducted untargeted UPLC/T-TOF-MS/MS metabolite profiling revealed a diverse array of secondary metabolites, including amino acids, flavonoids, alkaloids, nucleotides and carbohydrates, shedding light on the intricate chemical composition of CIR extract. The cytotoxic potential of the CIR extract was investigated in 15 human cell lines, which revealed that the CIR extract has a wide spectrum of antiproliferative activity with a high selectivity index, compared to that of MRC-5 cells. The most pronounced cytotoxic activity and selectivity index of the CIR extract was observed in the Hep-G2 and Panc-1 cells. Exploring the mode of action in Hep-G2 and Panc-1 cells revealed that the CIR extract substantially triggers the arrest of the cell cycle in the S phase, but also early and late apoptosis. These findings were further demonstrated by the notable alternation in the expression of pro-apoptotic and anti-apoptotic genes in both cell lines. Furthermore, our study revealed the antioxidant capacity of CIR extract, which displays a considerable total antioxidant capacity, together with notable free radicals and metal scavenging activities. Insights into anti-inflammatory activity, our results indicated that the CIR extract displays the ability to modulate inflammatory genes (IL-1b, IL-6, TNF-α), but also exhibits a pronounced selectivity towards COX-2 activity, suggesting its significant implications for the treatment of inflammatory and oxidative stress-related conditions. Finally, our *in silico* coupling studies further demonstrated the anti-inflammatory potential of the CIR extract and unveiled the observed selectivity towards cyclooxygenases 1 & 2. Collectively, these findings underscore the antitumor value of CIR extract as a natural-based therapeutic agent with multifaceted pharmacological benefits, including antioxidant, anti-inflammatory, and antiproliferative activities. Our results indicated that the CIR extract could interfere with key cellular processes including cell cycle, apoptosis, oxidative stress, and inflammatory pathways, which warrants further explorations of its applications in inflammatory conditions and cancer treatment.

## Data availability

Data supporting the results reported in this manuscript are included in this article. The raw data supporting the conclusions of this article will be made available by the authors without any undue reservation.

## Author contributions

Conceptualization, A. R. A., N. H., D. I. M., T. M. I. A., I. A. A. I., G. A. B., and E. M. S.; methodology, A. R. A., N. H., D. I. M., H. H. A. N., T. M. I. A., A. A., I. A. A. I., A. H. F., G. A. B., and E. M. S.; software N. H., D. I. M., T. M. I. A., A. A., I. A. A. I., A. H. F., and E. M. S.; validation, A. R. A., H. H. A. N., I. A. A. I., A. H. F., G. A. B., and E. M. S.; formal analysis, A. R. A., N. H., D. I. M., T. M. I. A., G. A. B., and E. M. S.; investigation, D. I. M., A. A., I. A. A. I., A. H. F., G. A. B., and E. M. S.; resources, A. R. A., N. H., D. I. M., T. M. I. A., A. A., and E. M. S.; data curation, A. R. A., N. H., D. I. M., H. H. A. N., T. M. I. A., A. A., I. A. A. I., A. H. F., G. A. B., and E. M. S.; writing—original draft preparation, A. R. A., N. H., D. I. M., H. H. A. N., T. M. I. A., A. A., I. A. A. I., A. H. F., G. A. B., and E. M. S.; writing—review and editing, A. R. A., N. H., D. I. M., A. A., H. H. A. N., T. M. I. A., I. A. A. I., A. H. F., G. A. B., and E. M. S.; visualization, A. R. A, H. H. A. N., A. S. A, I. A. A. I., S. M. Y. M., I. M. A., and I. S.; supervision A. R. A., A. H. F., G. A. B., and E. M. S.; project administration, A. R. A., N. H., D. I. M., T. M. I. A., I. A. A. I., G. A. B., and E. M. S.; funding acquisition, A. R. A., D. I. M., T. M. I. A., and E. M. S. All authors have read and agreed to the published version of the manuscript.

## Conflicts of interest

The authors declare no conflict of interest.

## Supplementary Material

RA-014-D4RA02131B-s001
