# Supplementary material for: Unveiling the multifaceted antiproliferative efficacy of Cichorium endivia root extract by dual modulation of apoptotic and inflammatory genes, inducing cell cycle arrest, and targeting COX-2
Source: RSC Adv. 2024 Jun 17;14(27):19400–27. doi: 10.1039/d4ra02131b (PMC11182420; doi:10.1039/d4ra02131b)
Supplement: RA-014-D4RA02131B-s001 [file RA-014-D4RA02131B-s001.pdf]

Electronic Supplementary Material (ESI) for RSC Advances.
This journal is © The Royal Society of Chemistry 2024

Supplementary Information

Unveiling the Multifaceted Antiproliferative Efficacy of *Cichorium endivia* Root Extract by Dual Modulation of Apoptotic and Inflammatory Genes, Inducing Cell Cycle Arrest, and Targeting COX-2

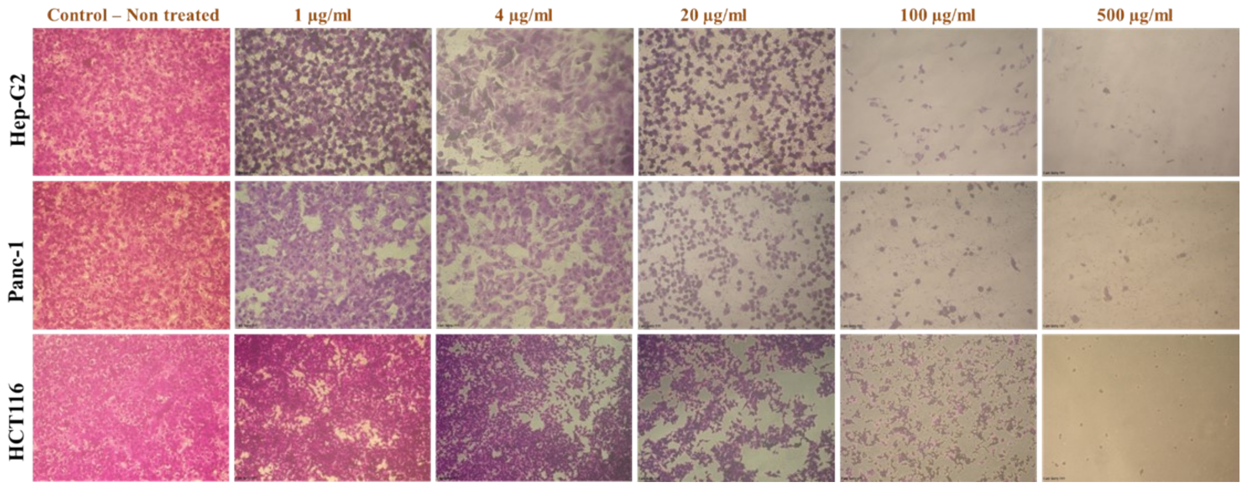

Figure S1: Evaluation of the morphological changes of HepG-2, Panc-1, and HCT-116 cells after treatment with CIR extract for 24 h at a concentration of 1, 4, 20, 100, and 500 µg/mL concentration.

.

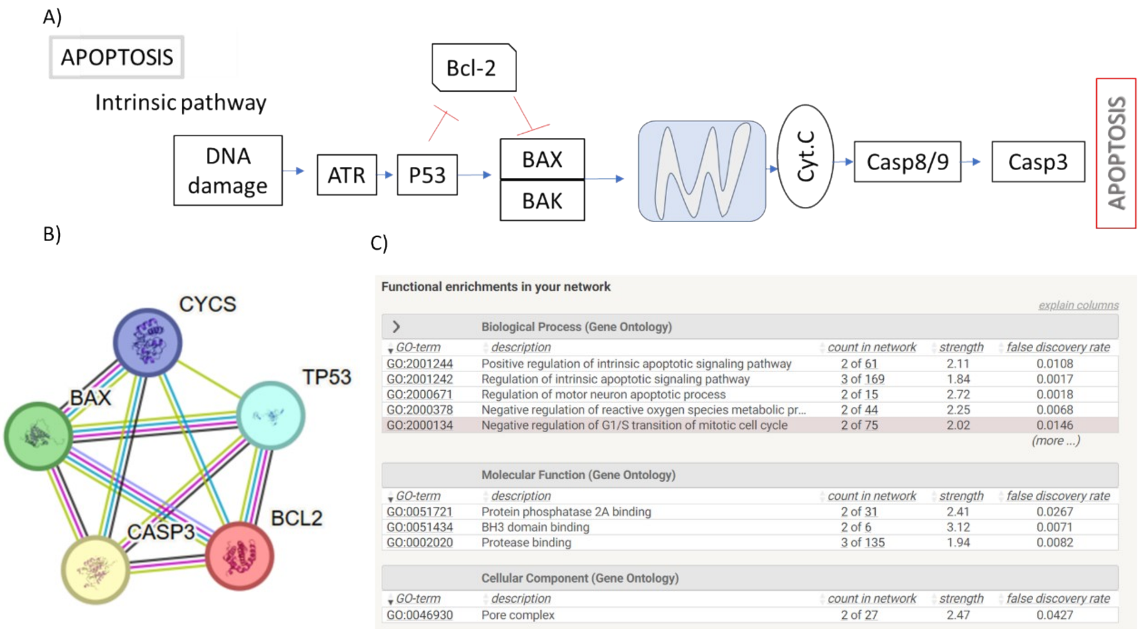

Biological Process (Gene Ontology)

GO-term	description	count in network	strength	false discovery rate
GO:2001244	Positive regulation of intrinsic apoptotic signaling pathway	2 of 61	2.11	0.0108
GO:2001242	Regulation of intrinsic apoptotic signaling pathway	3 of 169	1.84	0.0017
GO:2000671	Regulation of motor neuron apoptotic process	2 of 15	2.72	0.0018
GO:2000378	Negative regulation of reactive oxygen species metabolic pr...	2 of 44	2.25	0.0068
GO:2000134	Negative regulation of G1/S transition of mitotic cell cycle	2 of 75	2.02	0.0146

(more ...)

Molecular Function (Gene Ontology)

GO-term	description	count in network	strength	false discovery rate
GO:0051721	Protein phosphatase 2A binding	2 of 31	2.41	0.0267
GO:0051434	BH3 domain binding	2 of 6	3.12	0.0071
GO:0002020	Protease binding	3 of 135	1.94	0.0082

Cellular Component (Gene Ontology)

GO-term	description	count in network	strength	false discovery rate
GO:0046930	Pore complex	2 of 27	2.47	0.0427

Figure S2: A) KEGG analysis of the apoptosis pathway highlighting the genes playing key roles in the intrinsic apoptosis pathway (Casp3, Bax, Bcl-2, P53, and CYC genes), image reproduced from KEGG PATHWAY: Apoptosis - Reference pathway and KEGG PATHWAY: Apoptosis - multiple species - Reference pathway and Hendrata et al., 2016 (1, 2). **B)** Protein- protein interaction network for the investigated genes involved in apoptosis. Nodes represent proteins, and edges denote predicted associations. The colored lines indicate different types of evidence, with red representing fusion evidence, light blue representing database evidence, green representing neighborhood evidence, blue representing cooccurrence evidence, purple representing experimental evidence, black representing coexpression evidence, and yellow representing text mining evidence, interaction scores are detailed in supplementary **table S5**. STRING analysis (3). C) **Gene ontology and Functional enrichment** of the implicated proteins with the respective code of the involved processes (accessed on 15th February 2024, String Database) (3).

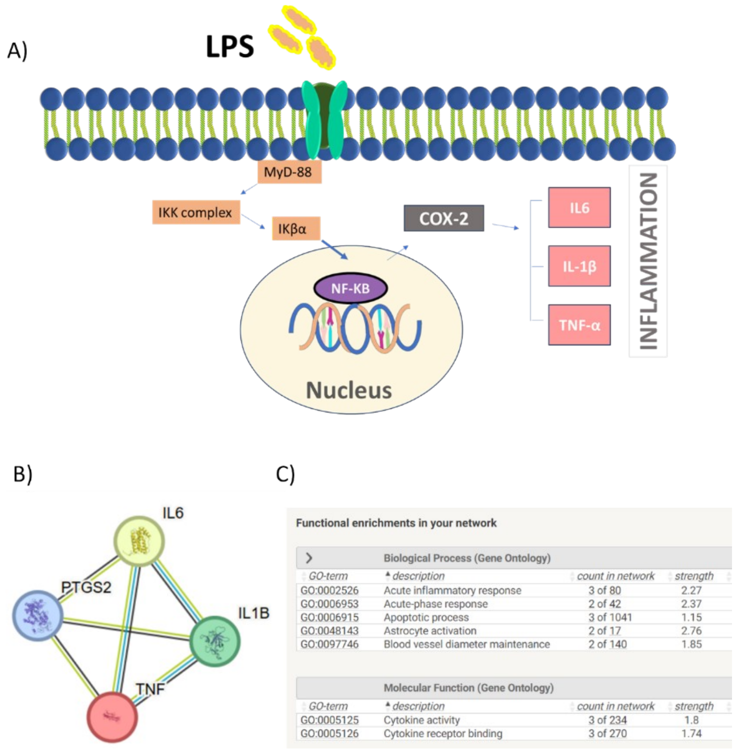

GO-term	description	count in network	strength
**Biological Process (Gene Ontology)**			
GO:0002526	Acute inflammatory response	3 of 80	2.27
GO:0006953	Acute-phase response	2 of 42	2.37
GO:0006915	Apoptotic process	3 of 1041	1.15
GO:0048143	Astrocyte activation	2 of 17	2.76
GO:0097746	Blood vessel diameter maintenance	2 of 140	1.85
**Molecular Function (Gene Ontology)**			
GO:0005125	Cytokine activity	3 of 234	1.8
GO:0005126	Cytokine receptor binding	3 of 270	1.74

Figure S3: IL-1b, IL-6 and TNF-*α* as selected inflammatory markers associated with the COX2 mediator, as shown by the KEGG pathway and illustrated by Khan et al., 2012 and Arulselvan et al., 2016 **(1, 4, 5). B)** Protein- protein interaction network for the investigated proteins involved in the inflammatory process. Nodes represent proteins, and edges denote predicted associations. The colored lines indicate different types of evidence, with red representing fusion evidence, light blue representing database evidence, green representing neighborhood evidence, blue representing co-occurrence evidence, purple representing experimental evidence, black representing co-expression evidence and yellow representing text mining evidence, interaction scores are detailed in supplementary **table S4**. STRING analysis (3). C) **Gene ontology and functional enrichment** of the involved proteins with the respective code of the implicated processes (accessed on 15th February 2024, String Database) (3).

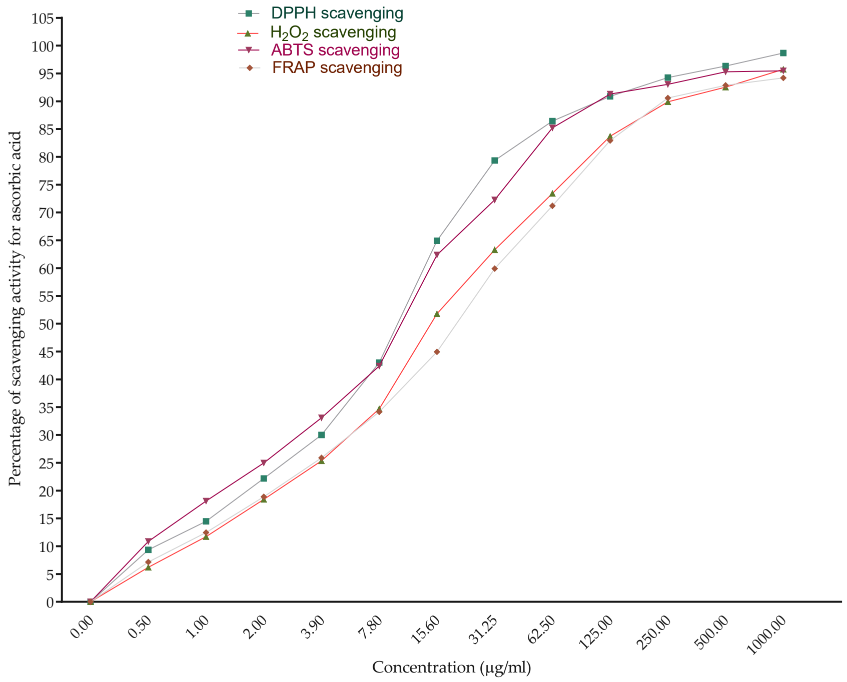

Figure S4: The dose-dependent scavenging activity of ascorbic acid against the DPPH radical, the H_2O_2 radical, the ABTS radical and FRAP.

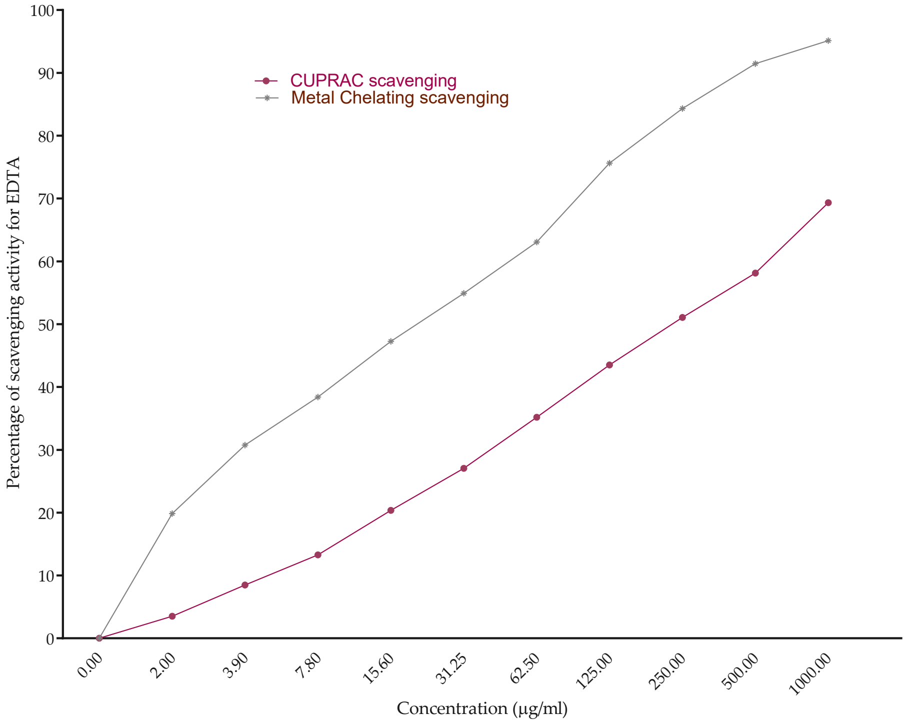

Figure S5: Dose-dependent scavenging activity of EDTA in relation to CURPAC and metal chelating activity.

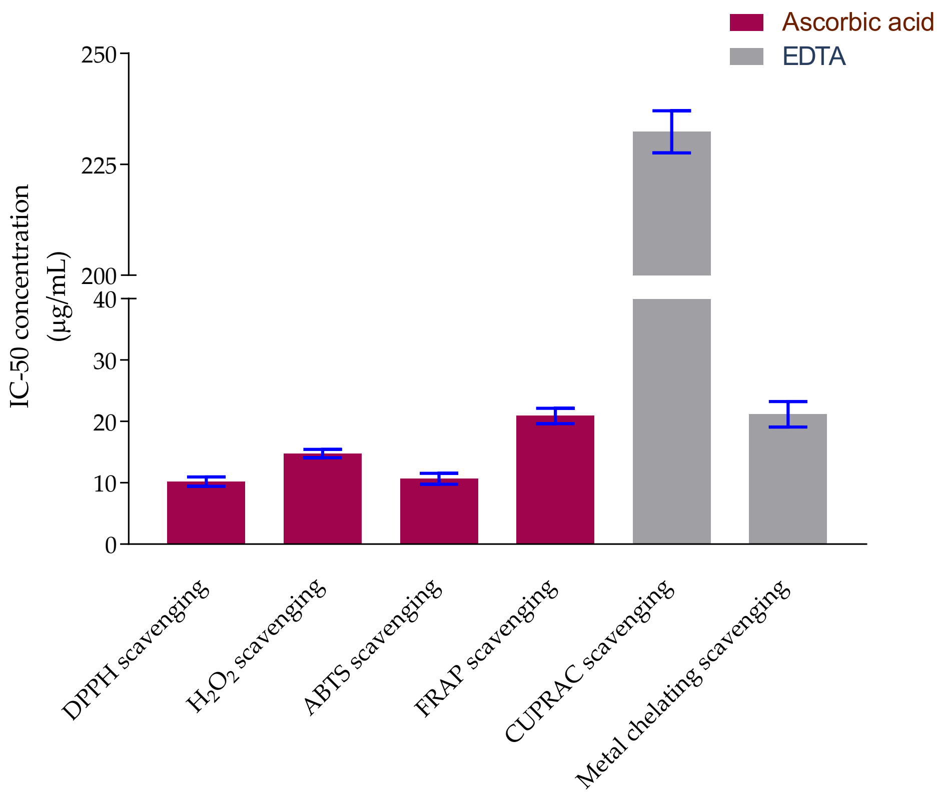

Figure S6: Representative diagram for the IC_{50} values of ascorbic acid and EDTA in respect of DPPH, H_2O_2, ABTS, FRAP, CURPAC and metal-chelating scavenging assays. Data were expressed as mean ± S.D in triplicate.

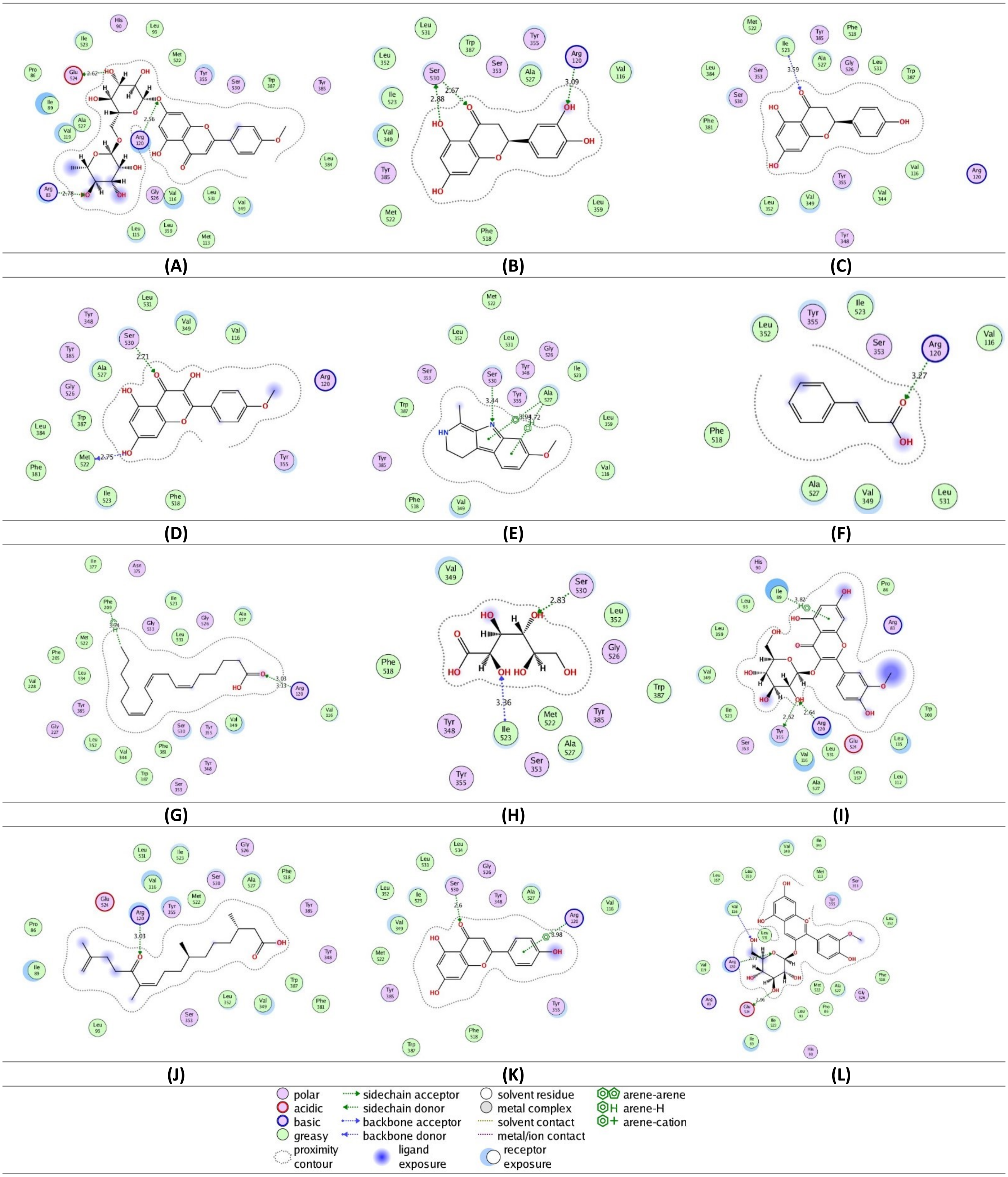

Figure S7: 2D representation of the binding mode of selected secondary metabolites identified in the CIR extract to the binding cavity of the COX-1 protein (PDB code: *1eqg*). **(A)** Acacetin-7-O-rutinoside; **(B)** 3', 4', 5, 7-tetrahydroxy-flavanone; **(C)** Naringenin; **(D)** 3, 5, 7-trihydroxy-4'-methoxy-flavone; **(E)** Harmaline; **(F)**

trans-Cinnamate; **(G)** γ-Linolenic acid; **(H)** Gluconate; **(I)** Isorhamnetin-3-O-glucoside; **(J)** 12-Oxo-10,15(Z)-Phytodienoic acid; **(K)** Apigenin; **(L)** Peonidine-3-O-glucoside.

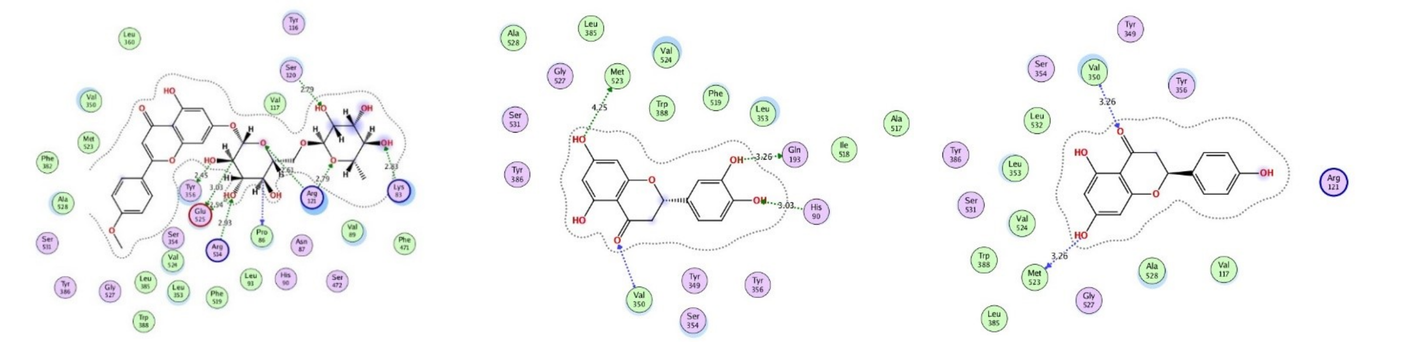

(A) (B) (C)

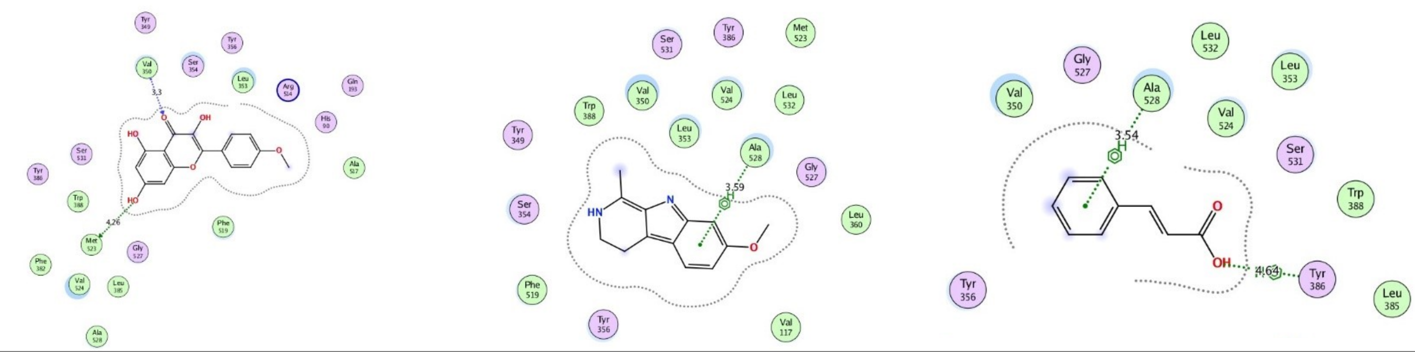

(D) (E) (F)

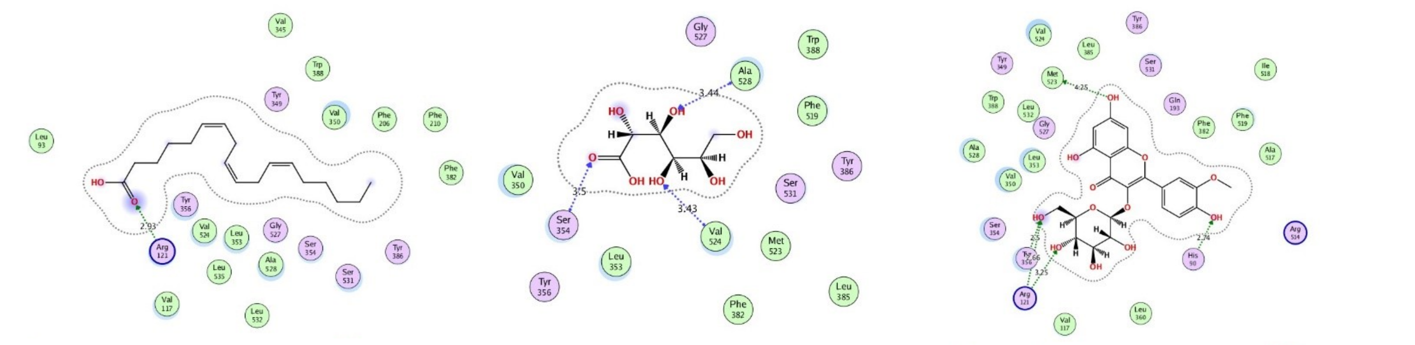

(G) (H) (I)

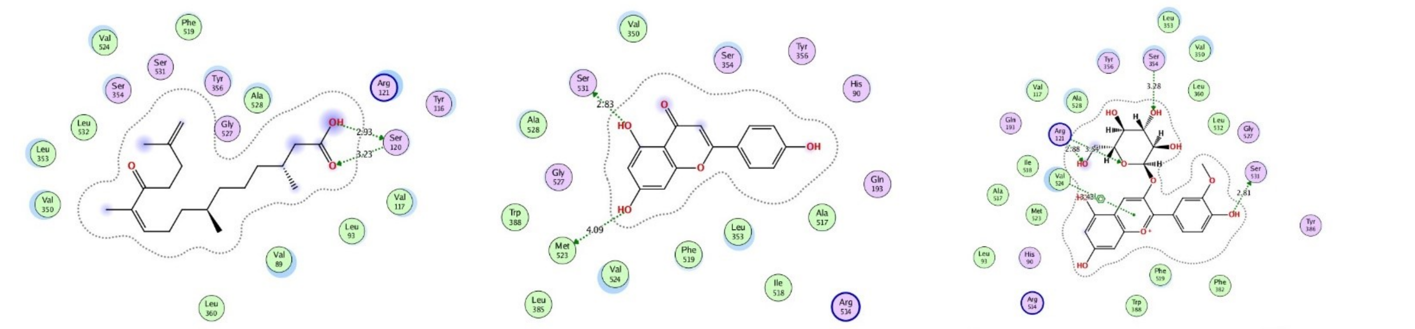

(J) (K) (L)

Figure S8: 2D representation of the binding mode of selected secondary metabolites identified in the CIR extract to the binding cavity of the COX-2 protein (PDB code: *4ph9*). **(A)** Acacetin-7-O-rutinoside; **(B)** 3', 4', 5, 7-tetrahydroxy-flavanone; **(C)** Naringenin; **(D)** 3, 5, 7-trihydroxy-4'-methoxy-flavone; **(E)** Harmaline; **(F)**

trans-Cinnamate; **(G)** γ-Linolenic acid; **(H)** Gluconate; **(I)** Isorhamnetin-3-O-glucoside; **(J)** 12-Oxo-10,15(Z)-Phytodienoic acid; **(K)** Apigenin; **(L)** Peonidine-3-O-glucoside.

Table S1: The list of gene primers used for the apoptotic and anti-inflammatory study.

Primer	Sequence	Reference
**Bax**	F 5′- TCAGGATGCGTCCACCAAGAAG -3′,	[26]
	R 5'- TGTGTCCACGGCGGCAATCATC-3'	
**Bcl2**	F 5′- ATCGCCCTGTGGATGACTGAGT -3′	[27]
	R 5'- GCCAGGAGAAATCAAACAGAGGC -3'	
**Casp3**	F 5′- GGAAGCGAATCAATGGACTCTGG-3′	[25]
	R 5'- GCATCGACATCTGTACCAGACC-3'	
**p53**	F 5′-CCTCAGCATCTTATCCGAGTGG -3′	[28]
	R 5'-TGGATGGTGGTACAGTCAGAGC -3'	
**CYC**	F 5′-AAGGGAGGCAAGCACAAGACTG -3′	[29]
	R 5'-CTCCATCAGTGTATCCTCTCCC -3'	
**GAPDH**	F 5′- GTCTCCTCTGACTTCAACAGCG-3′	[30]
	R 5′- ACCACCCTGTTGCTGTAGCCAA-3′	
**IL-1β**	F 5′-CCACAGACCTTCCAGGAGAATG -3′	[31]
	R 5'-GTGCAGTTCAGTGATCGTACAGG -3'	
**IL-6**	F 5′-AGACAGCCACTCACCTCTTCAG -3′	[32]
	R 5'-TTCTGCCAGTGCCTCTTTGCTG -3'	
**TNF-α**	F 5′-CTCTTCTGCCTGCTGCACTTTG -3′	[33]
	R 5'-ATGGGCTACAGGCTTGTCACTC -3'	

Table S2. The list of total nutrients detected in the CIR extract.

Nutrient	Concentration
Total saponin content	18.37 ± 0.121 mg AE/ g
Ash	1.59% ± 0.013
Total tannins	2.26± 0.103 mg/mL
Total proteins	48.11 ± 2.714 mg/mL
Total lipids	3.66 ± 0.290 mg/g
Total carbohydrates	1.45 ± 0.063 mg/g
Total alkaloids	163.82 ± 9.66 μg/g
Total phenolics	55.708 ± 1.91 mg/mL
Flavonoids	106.48 ± 4.17 mg/mL

Table S3: The list of metabolites detected in the *C. endivia* extract by UPLC/T-TOF–MS/MS spectrometer in positive ionization mode.

Title	RT (min)	Precursor (*m/z*)	Area	Error (PPM)	Adduct	Reference (*m/z*)	Formula	Ontology
**Acacetin-7-O-rutinoside**	24.031	593.2783	12237822	0.1	[M+H]+	593.18646	C28H32O14	Flavonoid-7-O-glycosides
**3' 4' 5 7-tetrahydroxyflavanone**	5.271	289.163	7469832	0.8	[M+H]+	289.07068	C15H12O6	Flavanones
**Naringenin**	7.527	273.1644	4594841	13.8	[M+H]+	273.07574	C15H12O5	Flavanones
**N,N-Dimethylglycine**	1.053	104.1062	4379100	3.9	[M+H]+	104.0706	C4H9NO2	Alpha amino acids
**3 5 7-trihydroxy-4'-methoxyflavone**	19.662	301.1429	3023031	0.6	[M+H]+	301.07068	C16H12O6	Flavonols
**12-Oxo-10,15(Z)-Phytodienoic Acid**	1.117	293.0635	1886257	-1.2	[M+H]+	293.21112	C18H28O3	Prostaglandins and related compounds
**Trigonelline**	1.142	138.0552	1573969	-0.1	[M+H]+	138.05496	C7H7NO2	Alkaloids and derivatives
Harmaline	5.063	215.1256	1403891	0.1	[M+H]+	215.11789	C13H14N2O	Harmala alkaloids
**trans-Cinnamate**	22.581	149.0246	1220626	-1.9	[M+H]+	149.05971	C9H8O2	Cinnamic acids
Isorhamnetin-3-O-glucoside	6.793	479.12	1024418	0.8	[M+H]+	479.11841	C22H22O12	Flavonoid-3-O-glycosides
**2'-Deoxycytidine**	19.925	228.2302	800974	12	[M+H]+	228.09789	C9H13N3O4	Pyrimidine 2'-deoxyribonucleosides
**DL-5-Hydroxylysine**	1.181	163.058	673049	12.8	[M+H]+	163.10771	C6H14N2O3	Alpha amino acids
**L-Asparagine**	1.298	133.0507	512950	-4.8	[M+H]+	133.06078	C4H8N2O3	Asparagine and derivatives
**Adenosine**	2.407733	268.1045	464233	0.8	[M+H]+	268.10403	C10H13N5O4	Purine nucleosides
**Apigenin**	11.05395	271.0616	459700	0.6	[M+H]+	271.06009	C15H10O5	Flavones
**Glycerophosphate(2)**	5.4784	173.0805	441894	1.8	[M+H]+	173.02095	C3H9O6P	Glycerophosphates
**Peonidine-3-O-glucoside chloride**	7.563967	463.1239	408484	-0.8	[M]+	463.12292	C22H23O11	Anthocyanidin-3-O-glycosides
**4-AMINOPHENOL**	0.8988166	110.0085	405899	0.7	[M+H]+	110.06004	C6H7NO	Aniline and substituted anilines
**D-Alloisoleucine**	1.39045	132.1012	325745	2.6	[M+H]+	132.1019	C6H13NO2	Isoleucine and

								derivatives
**3'-METHOXY-4',5,7-TRIHYDROXYFLAVONOL**	9.096884	317.0655	222116	1.9	[M+H]+	317.06558	C16H12O7	Flavonols
**(+-)-Taxifolin**	5.258417	305.1354	201960	3.9	[M+H]+	305.06558	C15H12O7	Flavanonols
**L-5-Oxoproline**	1.647283	130.0496	186844	0	[M+H]+	130.04987	C5H7NO3	Alpha amino acids and derivatives
**L-Valine**	1.2073	118.0861	177176	0.8	[M+H]+	118.08626	C5H11NO2	Valine and derivatives
**L-(-)-Phenylalanine**	2.061083	166.0833	162887	12.3	[M+H]+	166.08626	C9H11NO2	Phenylalanine and derivatives
**Malvidin-3-O-glucoside chloride**	7.82195	493.1365	155355	-0.4	[M]+	493.13351	C23H25O12	Anthocyanidin-3-O-glycosides
**L-PROLINE**	1.155467	116.069	143491	9.8	[M+H]+	116.0706	C5H9NO2	Proline and derivatives
**Daphnetin**	7.945617	179.1109	107743	-9.4	[M+H]+	179.03389	C9H6O4	7,8-dihydroxycoumarins
**2'-Deoxyadenosine 5'-monophosphate**	1.416783	332.1106	103832	-0.8	[M+H]+	332.07544	C10H14N5O6 P	Purine 2'-deoxyribonucleoside monophosphates
**S-Adenosyl-L-homocysteine**	10.01483	385.282	97268	-25.7	[M+H]+	385.12888	C14H20N6O5S	5'-deoxy-5'-thionucleosides
**Leupeptin hemisulfate salt**	21.78888	427.3107	94923	13.4	[M+H]+	427.30273	C20H38N6O4	Dipeptides
**D-(-)-Erythrose**	19.68733	121.0312	93365	-13.6	[M+H]+	121.04954	C4H8O4	Pentoses
**Histidinol**	2.3579	142.1605	86176	-6.6	[M+H]+	142.09749	C6H11N3O	Aralkylamines
**Pipecolate**	1.194133	130.1043	77334	-100.6	[M+H]+	130.08626	C6H11NO2	Alpha amino acids
**L-Glutamic acid**	1.155467	148.0596	71180	0.7	[M+H]+	148.06044	C5H9NO4	Glutamic acid and derivatives
**Xanthosine-5'-monophosphate**	10.74463	365.1946	69798	-0.7	[M+H]+	365.04929	C10H13N4O9 P	Purine ribonucleoside monophosphates
**Baicalein-7-O-glucuronide**	8.909567	447.092	66314	0.4	[M+H]+	447.09219	C21H18O11	Flavonoid-7-O-glucuronides
**Amantadine**	1.233633	152.0554	65059	13.9	[M+H]+	152.14337	C10H17N	Monoalkylamines
**S-Adenosyl-L-**	10.91645	399.1646	63754	1	[M+H]+	399.1445	C15H22N6O5S	5'-deoxy-5'-

**methionine**								thionucleosides
**N-Acetylneuraminate**	2.04875	310.1284	62994	-0.3	[M+H]+	310.11325	C11H19NO9	N-acylneuraminic acids
**NICOTINIC ACID**	26.83012	124.088	60233	0	[M+H]+	124.03931	C6H5NO2	Pyridinecarboxylic acids
**Caffeine**	15.3734	195.1351	59621	13.7	[M+H]+	195.08765	C8H10N4O2	Xanthines
**Glycerol-2-phosphate**	12.58503	173.0813	52992	0.4	[M+H]+	173.02095	C3H9O6P	Glycerophosphates
**3-FORMYLINDOLE**	8.1786	146.0563	48678	18.7	[M+H]+	146.06004	C9H7NO	Indoles
**Cytidine**	1.1298	244.0885	48328	11.6	[M+H]+	244.0928	C9H13N3O5	Pyrimidine nucleosides
**Peonidin-3,5-O-di-beta-glucopyranoside**	7.29415	625.1793	43936	-0.6	[M]+	625.17578	C28H33O16	Anthocyanidin-5-O-glycosides
**Ononin**	12.04923	431.1606	43353	14	[M+H]+	431.13367	C22H22O9	Isoflavonoid O-glycosides
**Piperazine**	1.065817	87.04267	41783	2.5	[M+H]+	87.09167	C4H10N2	Piperazines
**UROCANIC ACID**	8.647233	139.1116	41696	0.4	[M+H]+	139.0502	C6H6N2O2	Imidazolyl carboxylic acids and derivatives
**(-)-RIBOFLAVIN**	11.32927	377.2278	40152	3.4	[M+H]+	377.14557	C17H20N4O6	Flavins
**D-Glucosamine-6-phosphate**	1.142467	260.0589	37767	-7.5	[M+H]+	260.05298	C6H14NO8P	Hexose phosphates
**Resveratrol**	1.246967	229.1562	34568	-1.9	[M+H]+	229.08592	C14H12O3	Stilbenes
**PIPERIDINE**	1.053317	86.05891	32043	-0.6	[M+H]+	86.09643	C5H11N	Piperidines
**Quercetin-3-Arabinoside**	11.17945	435.2083	31917	-8.4	[M+H]+	435.09219	C20H18O11	Flavonoid-3-O-glycosides
**Luteolin-8-C-glucoside**	12.18622	449.1789	28702	-1.3	[M+H]+	449.10785	C21H20O11	Flavonoid 8-C-glycosides
**Adenine**	7.4168	136.0773	28654	0	[M+H]+	136.06177	C5H5N5	6-aminopurines
**DL-Cystathionine**	13.27617	223.1344	28309	-1.2	[M+H]+	223.07471	C7H14N2O4S	L-cysteine-S-conjugates
**Malvidin-3, 5-di-O-glucoside chloride**	21.14975	655.3069	27569	8.7	[M]+	655.18634	C29H35O17	Anthocyanidin-5-O-glycosides
**3-(4-HYDROXY-3-METHOXYPHENYL)PROP-2-ENOICACID**	1.592283	194.9686	26189	-4.2	[M+H]+	195.06519	C10H10O4	Hydroxycinnamic acids
**Apigenin 8-C-glucoside**	12.6967	433.1982	25978	-21.2	[M+H]+	433.11292	C21H20O10	Flavonoid 8-C-glycosides
**CHOLIC ACID**	7.391967	409.186	25082	1	[M+H]+	409.29486	C24H40O5	Trihydroxy bile acids,

								alcohols and derivatives
**Glycine-Betaine**	26.80512	118.0856	24893	7	[M+H]+	118.08626	C5H11NO2	Alpha amino acids
**Formononetin**	22.06138	269.0985	24469	6.3	[M+H]+	269.08084	C16H12O4	4'-O-methylisoflavones
**Histamine**	1.2073	112.0874	21528	-3.2	[M+H]+	112.08692	C5H9N3	2-arylethylamines
**Isoguvacine**	21.37923	128.1057	19693	0.5	[M+H]+	128.0706	C6H9NO2	Hydropyridines
**Pyridoxamine**	17.85027	169.1206	19583	11.8	[M+H]+	169.09715	C8H12N2O2	Pyridoxamine 5'-phosphates
**Gossypin**	4.9661	481.1261	15961	12.3	[M+H]+	481.09766	C21H20O13	Flavonoid-8-O-glycosides
**2-(4-ISOBUTYLPHENYL)PROPIONIC ACID**	11.22945	207.0997	14321	3.2	[M+H]+	207.13795	C13H18O2	Phenylpropanoic acids
**3,4-DIMETHOXYCINNAMIC ACID**	14.97175	209.1185	12223	7.9	[M+H]+	209.08084	C11H12O4	Coumaric acids and derivatives
**HYPOXANTHINE**	18.17042	137.1332	11300	-0.1	[M+H]+	137.04579	C5H4N4O	Hypoxanthines
**NICOTINAMIDE**	1.246967	123.0917	8499	1	[M+H]+	123.05529	C6H6N2O	Nicotinamides
**1,4-BENZOQUINONE**	8.7224	109.1027	7965	-11.4	[M+H]+	109.0284	C6H4O2	P-benzoquinones
**Cytidine-3',5'-cyclicmonophosphate**	6.656183	306.2336	7005	-7.8	[M+H]+	306.04855	C9H12N3O7P	Pentose phosphates

Table S4: The list of metabolites detected in the *C. endivia* extract by UPLC/T-TOF–MS/MS spectrometer in the negative ionization mode.

Title	RT (min)	Precursor (*m/z*)	Area	Error (PPM)	Adduct	Reference (*m/z*)	Formula	Ontology
**γ-Linolenic acid**	19.07118	277.2176	37943284	-0.9	[M-H]-	277.21732	C18H30O2	Lineolic acids and derivatives
**Gluconate**	0.9558	195.0495	5327352	6	[M-H]-	195.05103	C6H12O7	Medium-chain hydroxy acids and derivatives
**D-(-)-Quinic acid**	0.930466 7	191.0543	3485385	7.4	[M-H]-	191.05611	C7H12O6	Quinic acids and derivatives
**D-(+)-Malic acid**	0.8793	133.0128	3334704	6.6	[M-H]-	133.01425	C4H6O5	Beta hydroxy acids and derivatives
**MALEIC ACID**	0.8793	115.0034	637716	-0.6	[M-H]-	115.00368	C4H4O4	Dicarboxylic acids and derivatives
**3-Phospho-D-glycerate**	5.189083	185.1162	584261	8	[M-H]-	184.98566	C3H7O7P	Sugar acids and derivatives
**SUCCINIC ACID**	0.891966 6	117.0177	256807	6.5	[M-H]-	117.01933	C4H6O4	Dicarboxylic acids and derivatives
**3,4-Dihydroxymandelate**	4.526767	183.1042	251793	-6.7	[M-H]-	183.02989	C8H8O5	Catechols
**CITRACONIC ACID**	1.5926	128.9574	165623	10.1	[M-H]-	129.01933	C5H6O4	Methyl-branched fatty acids
**Glyceric acid**	0.9558	105.0182	80810	3.9	[M-H]-	105.01933	C3H6O4	Sugar acids and derivatives
**BETA-INDOLEACETIC ACID**	7.079134	174.0574	36304	-6.4	[M-H]-	174.05605	C10H9NO2	Indole-3-acetic acid derivatives
**Lactic acid**	0.8793	89.02464	35426	-3.2	[M-H]-	89.02441	C3H6O3	Alpha hydroxy acids and derivatives
**2-Isopropylmalic acid**	1.0318	175.0595	24760	4.2	[M-H]-	175.0612	C7H12O5	Hydroxy fatty acids
**UROCANIC ACID**	1.13345	137.0233	21241	0.6	[M-H]-	137.03564	C6H6N2O2	Imidazolyl carboxylic acids and derivatives
**Gibberelin A3**	6.071367	345.155	17337	0	[M-H]-	345.13437	C19H22O6	C19-gibberellin 6-

								carboxylic acids
**3-(4-HYDROXYPHENYL)PROP-2-ENOIC ACID**	1.225783	163.0344	6626	24.7	[M-H]-	163.04007	C9H8O3	Hydroxycinnamic acids
**L-beta-Homoisoleucine**	8.039416	144.0447	602921	0.6	[M-H]-	144.103	C7H15NO2	Beta amino acids and derivatives
**trans-4-Hydroxy-L-proline**	1.277117	130.0844	297606	13.2	[M-H]-	130.05096	C5H9NO3	Proline and derivatives
**L-5-Oxoproline**	0.9683	128.0341	114097	0	[M-H]-	128.03532	C5H7NO3	Alpha amino acids and derivatives
**5-AMINOVALERIC ACID**	1.172283	116.0693	72930	12	[M-H]-	116.0717	C5H11NO2	Delta amino acids and derivatives
**L-(-)-Phenylalanine**	1.878417	164.0694	57012	8.2	[M-H]-	164.0717	C9H11NO2	Phenylalanine and derivatives
**L-Glutamic acid**	0.9429666	146.0447	52690	1.8	[M-H]-	146.04588	C5H9NO4	Glutamic acid and derivatives
**L-(-)-Threonine**	1.121117	118.0477	15209	17.1	[M-H]-	118.05096	C4H9NO3	L-alpha-amino acids
**Esculin**	15.84418	339.201	203112	-1.5	[M-H]-	339.07214	C15H16O9	Coumarin glycosides
**Galactinol Dihydrate**	1.09545	341.1078	65945	0.4	[M-H]-	341.10895	C12H22O11	O-glycosyl compounds
**4-Methylsulfinylbutyl glucosinolate**	11.63257	436.1941	19156	6	[M-H]-	436.04114	C12H23NO10S3	Alkylglucosinolates
**E-3,4,5'-Trihydroxy-3'-glucopyranosylstilbene**	8.495566	405.1167	12901	6.5	[M-H]-	405.11911	C20H22O9	Stilbene glycosides
**Apigenin**	10.39897	269.0444	5452233	3.3	[M-H]-	269.04553	C15H10O5	Flavones
**Peonidine-3-O-glucoside chloride**	7.38995	461.1098	1011160	-0.8	[M-2H]-	461.1084	C22H23O11	Anthocyanidin-3-O-glycosides
**3 5 7-trihydroxy-4'-methoxyflavone**	9.94305	299.0498	658367	20.5	[M-H]-	299.05612	C16H12O6	Flavonols
**Petunidin-3-O-β-glucopyranoside**	7.079134	477.1029	634073	1.3	[M-2H]-	477.1033	C22H23O12	Anthocyanidin-3-O-glycosides
**Isorhamnetin-3-O-**	8.882717	477.1037	394143	-0.3	[M-H]-	477.10385	C22H22O12	Flavonoid-3-O-glycosides

**glucoside**								
**3'-METHOXY-4',5,7-TRIHYDROXYFLAVONOL**	9.035033	315.05	280182	1.8	[M-H]-	315.05103	C16H12O7	Flavonols
**Baicalein-7-O-glucuronide**	5.722217	445.078	220866	0.4	[M-H]-	445.07764	C21H18O11	Flavonoid-7-O-glucuronides
**Kaempferol-3-O-alpha-L-rhamnoside**	0.9429666	431.0771	114237	0.7	[M-H]-	431.09836	C21H20O10	Flavonoid-3-O-glycosides
**Isorhamnetin-3-O-rutinoside**	7.129967	623.1536	62631	11.9	[M-H]-	623.16174	C28H32O16	Flavonoid-3-O-glycosides
**Quercitrin**	1.198283	447.1186	42538	-8	[M-H]-	447.09329	C21H20O11	Flavonoid-3-O-glycosides
**Delphinidin-3-O-(6''-O-alpha-rhamnopyranosyl-beta-glucopyranoside)**	6.308517	609.1357	24937	15.7	[M-2H]-	609.14557	C27H31O16	Anthocyanidin-3-O-glycosides
**eriodictyol-7-O-glucoside**	0.9304667	449.0646	22343	1.1	[M-H]-	449.10895	C21H22O11	Flavonoid-7-O-glycosides
**Daidzein-8-C-glucoside**	5.453233	415.1634	15651	-5.4	[M-H]-	415.10345	C21H20O9	Isoflavonoid C-glycosides
**Acacetin-7-O-rutinoside**	0.9558	590.9988	12970	4	[M-H]-	591.17194	C28H32O14	Flavonoid-7-O-glycosides
**Rhoifolin**	8.596566	577.1898	11024	0.7	[M-H]-	577.15625	C27H30O14	Flavonoid-7-O-glycosides
**Uridine**	1.225783	243.0622	498791	-0.5	[M-H]-	243.06226	C9H12N2O6	Pyrimidine nucleosides
**2'-Deoxyinosine 5'-monophosphate**	10.09732	330.9835	203250	-9.8	[M-H]-	331.04492	C10H13N4O7P	Purine 2'-deoxyribonucleoside monophosphates
**2'-Deoxyuridine-5'-monophosphate**	6.5295	307.1383	159510	4.5	[M-H]-	307.03366	C9H13N2O8P	Pyrimidine 2'-deoxyribonucleoside monophosphates
**Guanosine**	1.225783	282.0826	103115	4.7	[M-H]-	282.08438	C10H13N5O5	Purine nucleosides
**Inosine-5'-monophosphate**	4.564267	347.1699	97226	-0.1	[M-H]-	347.03983	C10H13N4O8P	Purine ribonucleoside monophosphates
**2'-Deoxyuridine**	18.69637	227.2016	87980	-1	[M-H]-	227.06735	C9H12N2O5	Pyrimidine 2'-

								deoxyribonucleosides
**Adenine**	1.4091	134.0461	83209	0.9	[M-H]-	134.04723	C5H5N5	6-aminopurines
**5-Aminoimidazole-4-carboxamide-1-ribofuranosyl 5'-monophosphate**	15.9815	337.2069	54558	-1.9	[M-H]-	337.05548	C9H15N4O8P	1-ribosyl-imidazolecarboxamides
**Thymidine**	7.7971	241.1083	22253	-0.1	[M-H]-	241.08299	C10H14N2O5	Pyrimidine 2'-deoxyribonucleosides
**Inosine**	1.225783	267.0903	22131	-28.1	[M-H]-	267.07349	C10H12N4O5	Purine nucleosides
**Isopentenyladenine**	10.19798	202.0499	10938	1.1	[M-H]-	202.10982	C10H13N5	6-alkylaminopurines
**Sodium Deoxycholate**	5.684216	391.1569	159076	8.3	[M-H]-	391.28537	C24H40O4	Dihydroxy bile acids, alcohols and derivatives
**Mucate**	9.29685	209.1147	61892	11.7	[M-H]-	209.03029	C6H10O8	Glucuronic acid derivatives
**Xanthine**	8.520733	151.0411	61529	-1.2	[M-H]-	151.02615	C5H4N4O2	Xanthines
**1-Myristoyl-2-hydroxy-sn-glycero-3-phosphate**	16.44898	381.2278	34340	7	[M-H]-	381.20477	C17H35O7P	1-acylglycerol-3-phosphates
**GAMMA-TERPINENE**	6.5295	135.0434	32088	2.1	[M-H]-	135.11792	C10H16	Branched unsaturated hydrocarbons
**L-Homocarnosine**	9.772833	239.0674	25767	0	[M-H]-	239.11496	C10H16N4O3	Hybrid peptides

Table S5: The interaction scores of the protein-protein interaction network for the investigated genes involved in the inflammatory pathway.

node1	node2	node1 accession	node2 accession	node1 annotation	node2 annotation	score
**IL1B**	IL6	ENSP00000263341	ENSP00000385675	*Interleukin-1 beta; Potent proinflammatory cytokine. Initially discovered as the major endogenous pyrogen, induces prostaglandin synthesis, neutrophil influx and activation, T-cell activation and cytokine production, B- cell activation and antibody production, and fibroblast proliferation and collagen production. Promotes Th17 differentiation of T-cells. Synergizes with IL12/interleukin-12 to induce IFNG synthesis from T- helper 1 (Th1) cells.*	*Interleukin-6; Cytokine with a wide variety of biological functions. It is a potent inducer of the acute phase response. Plays an essential role in the final differentiation of B-cells into Ig-secreting cells Involved in lymphocyte and monocyte differentiation. Acts on B-cells, T-cells, hepatocytes, hematopoietic progenitor cells and cells of the CNS. Required for the generation of T(H)17 cells. Also acts as a myokine. It is discharged into the bloodstream after muscle contraction and acts to increase the breakdown of fats and to improve insulin resistance. It induces myeloma and plasm [...]*	0.996
**IL1B**	PTGS2	ENSP00000263341	ENSP00000356438	*Interleukin-1 beta; Potent proinflammatory cytokine. Initially discovered as the major endogenous pyrogen, induces prostaglandin synthesis, neutrophil influx and activation, T-cell activation and cytokine production, B- cell activation and antibody production, and fibroblast proliferation and collagen production. Promotes Th17 differentiation of T-cells. Synergizes with IL12/interleukin-12 to induce IFNG synthesis from T- helper 1 (Th1) cells.*	*Prostaglandin G/H synthase 2; Converts arachidonate to prostaglandin H2 (PGH2), a committed step in prostanoid synthesis. Constitutively expressed in some tissues in physiological conditions, such as the endothelium, kidney and brain, and in pathological conditions, such as in cancer. PTGS2 is responsible for production of inflammatory prostaglandins. Up-regulation of PTGS2 is also associated with increased cell adhesion, phenotypic changes, resistance to apoptosis and tumor angiogenesis. In cancer cells, PTGS2 is a key step in the production of prostaglandin E2 (PGE2), which plays imp [...]*	0.974
**IL1B**	TNF	ENSP00000263341	ENSP00000398698	*Interleukin-1 beta; Potent proinflammatory cytokine. Initially discovered as the major endogenous pyrogen, induces prostaglandin synthesis, neutrophil influx and activation, T-cell activation and cytokine production, B- cell activation and antibody production, and fibroblast proliferation and collagen production. Promotes Th17 differentiation of T-cells. Synergizes with IL12/interleukin-12 to induce IFNG synthesis from T- helper 1 (Th1) cells.*	*Tumor necrosis factor, membrane form; Cytokine that binds to TNFRSF1A/TNFR1 and TNFRSF1B/TNFBR. It is mainly secreted by macrophages and can induce cell death of certain tumor cell lines. It is potent pyrogen causing fever by direct action or by stimulation of interleukin-1 secretion and is implicated in the induction of cachexia, Under certain conditions it can stimulate cell proliferation and induce cell differentiation. Impairs regulatory T- cells (Treg) function in individuals with rheumatoid arthritis via FOXP3 dephosphorylation. Upregulates the expression of protein phosphatase 1 [...]*	0.998
**IL6**	IL1B	ENSP00000385675	ENSP00000263341	*Interleukin-6; Cytokine with a wide variety of biological functions. It is a potent inducer of the acute phase response. Plays an essential role in the final differentiation of B-cells into Ig-secreting cells Involved in lymphocyte and monocyte differentiation. Acts on B-cells, T-cells, hepatocytes, hematopoietic progenitor cells and cells of the CNS. Required for the generation of T(H)17 cells. Also acts as a myokine. It is discharged into the bloodstream after muscle contraction and acts to increase the breakdown of fats and to improve insulin resistance. It induces myeloma and plasm [...]*	*Interleukin-1 beta; Potent proinflammatory cytokine. Initially discovered as the major endogenous pyrogen, induces prostaglandin synthesis, neutrophil influx and activation, T-cell activation and cytokine production, B- cell activation and antibody production, and fibroblast proliferation and collagen production. Promotes Th17 differentiation of T-cells. Synergizes with IL12/interleukin-12 to induce IFNG synthesis from T- helper 1 (Th1) cells.*	0.996
**IL6**	PTGS2	ENSP00000385675	ENSP00000356438	*Interleukin-6; Cytokine with a wide variety of biological functions. It is a potent inducer of the acute phase response. Plays an essential role in the final differentiation of B-cells into Ig-secreting cells Involved in lymphocyte and monocyte differentiation. Acts on B-cells, T-cells, hepatocytes, hematopoietic progenitor cells and cells of the CNS. Required for the generation of T(H)17 cells. Also acts as a myokine. It is discharged into the bloodstream after muscle contraction and acts to increase the breakdown of fats and to improve insulin resistance. It induces myeloma and plasm [...]*	*Prostaglandin G/H synthase 2; Converts arachidonate to prostaglandin H2 (PGH2), a committed step in prostanoid synthesis. Constitutively expressed in some tissues in physiological conditions, such as the endothelium, kidney and brain, and in pathological conditions, such as in cancer. PTGS2 is responsible for production of inflammatory prostaglandins. Up-regulation of PTGS2 is also associated with increased cell adhesion, phenotypic changes, resistance to apoptosis and tumor angiogenesis. In cancer cells, PTGS2 is a key step in the production of prostaglandin E2 (PGE2), which plays imp [...]*	0.96
**IL6**	TNF	ENSP00000385675	ENSP00000398698	*Interleukin-6; Cytokine with a wide variety of biological functions. It is a potent inducer of the acute phase response. Plays an essential role in the final differentiation of B-cells into Ig-secreting cells Involved in lymphocyte and monocyte differentiation. Acts on B-cells, T-cells, hepatocytes, hematopoietic progenitor cells and cells of the CNS. Required for the generation of T(H)17 cells. Also acts as a myokine. It is discharged into the bloodstream after muscle contraction and acts to increase the breakdown of fats and to improve insulin resistance. It induces myeloma and plasm [...]*	*Tumor necrosis factor, membrane form; Cytokine that binds to TNFRSF1A/TNFR1 and TNFRSF1B/TNFBR. It is mainly secreted by macrophages and can induce cell death of certain tumor cell lines. It is potent pyrogen causing fever by direct action or by stimulation of interleukin-1 secretion and is implicated in the induction of cachexia, Under certain conditions it can stimulate cell proliferation and induce cell differentiation. Impairs regulatory T- cells (Treg) function in individuals with rheumatoid arthritis via FOXP3 dephosphorylation. Upregulates the expression of protein phosphatase 1 [...]*	0.994
**PTGS2**	IL1B	ENSP00000356438	ENSP00000263341	*Prostaglandin G/H synthase 2; Converts arachidonate to prostaglandin H2 (PGH2), a committed step in prostanoid synthesis. Constitutively expressed in some tissues in physiological conditions, such as the endothelium, kidney and brain, and in pathological conditions, such as in cancer. PTGS2 is responsible for production of inflammatory prostaglandins. Up-regulation of PTGS2 is also associated with increased cell adhesion, phenotypic changes, resistance to apoptosis and tumor angiogenesis. In cancer cells, PTGS2 is a key step in the production of prostaglandin E2 (PGE2),*	*Interleukin-1 beta; Potent proinflammatory cytokine. Initially discovered as the major endogenous pyrogen, induces prostaglandin synthesis, neutrophil influx and activation, T-cell activation and cytokine production, B- cell activation and antibody production, and fibroblast proliferation and collagen production. Promotes Th17 differentiation of T-cells. Synergizes with IL12/interleukin-12 to induce IFNG synthesis from T- helper 1 (Th1) cells.*	0.974

				which plays imp [...]		
**PTGS2**	IL6	ENSP00000356438	ENSP00000385675	*Prostaglandin G/H synthase 2; Converts arachidonate to prostaglandin H2 (PGH2), a committed step in prostanoid synthesis. Constitutively expressed in some tissues in physiological conditions, such as the endothelium, kidney and brain, and in pathological conditions, such as in cancer. PTGS2 is responsible for production of inflammatory prostaglandins. Up-regulation of PTGS2 is also associated with increased cell adhesion, phenotypic changes, resistance to apoptosis and tumor angiogenesis. In cancer cells, PTGS2 is a key step in the production of prostaglandin E2 (PGE2), which plays imp [...]*	*Interleukin-6; Cytokine with a wide variety of biological functions. It is a potent inducer of the acute phase response. Plays an essential role in the final differentiation of B-cells into Ig-secreting cells Involved in lymphocyte and monocyte differentiation. Acts on B-cells, T-cells, hepatocytes, hematopoietic progenitor cells and cells of the CNS. Required for the generation of T(H)17 cells. Also acts as a myokine. It is discharged into the bloodstream after muscle contraction and acts to increase the breakdown of fats and to improve insulin resistance. It induces myeloma and plasm [...]*	0.96
**PTGS2**	TNF	ENSP00000356438	ENSP00000398698	*Prostaglandin G/H synthase 2; Converts arachidonate to prostaglandin H2 (PGH2), a committed step in prostanoid synthesis. Constitutively expressed in some tissues in physiological conditions, such as the endothelium, kidney and brain, and in pathological conditions, such as in cancer. PTGS2 is responsible for production of inflammatory prostaglandins. Up-regulation of PTGS2 is also associated with increased cell adhesion, phenotypic changes, resistance to apoptosis and tumor angiogenesis. In cancer cells, PTGS2 is a key step in the production of prostaglandin E2 (PGE2), which plays imp [...]*	*Tumor necrosis factor, membrane form; Cytokine that binds to TNFRSF1A/TNFR1 and TNFRSF1B/TNFBR. It is mainly secreted by macrophages and can induce cell death of certain tumor cell lines. It is potent pyrogen causing fever by direct action or by stimulation of interleukin-1 secretion and is implicated in the induction of cachexia, Under certain conditions it can stimulate cell proliferation and induce cell differentiation. Impairs regulatory T- cells (Treg) function in individuals with rheumatoid arthritis via FOXP3 dephosphorylation. Upregulates the expression of protein phosphatase 1 [...]*	0.916
**TNF**	IL1B	ENSP00000398698	ENSP00000263341	*Tumor necrosis factor, membrane form; Cytokine that binds to TNFRSF1A/TNFR1 and TNFRSF1B/TNFBR. It is mainly secreted by macrophages and can induce cell death of certain tumor cell lines. It is potent pyrogen causing fever by direct action or by stimulation of interleukin-1 secretion and is implicated in the induction of cachexia, Under certain conditions it can stimulate cell proliferation and induce cell differentiation. Impairs regulatory T-cells (Treg) function in individuals with rheumatoid arthritis via FOXP3 dephosphorylation. Upregulates the expression of protein phosphatase 1 [...]*	*Interleukin-1 beta; Potent proinflammatory cytokine. Initially discovered as the major endogenous pyrogen, induces prostaglandin synthesis, neutrophil influx and activation, T-cell activation and cytokine production, B- cell activation and antibody production, and fibroblast proliferation and collagen production. Promotes Th17 differentiation of T-cells. Synergizes with IL12/interleukin-12 to induce IFNG synthesis from T- helper 1 (Th1) cells.*	0.998
**TNF**	IL6	ENSP00000398698	ENSP00000385675	*Tumor necrosis factor, membrane form; Cytokine that binds to TNFRSF1A/TNFR1 and TNFRSF1B/TNFBR. It is mainly secreted by macrophages and can induce cell death of certain tumor cell lines. It is potent pyrogen causing fever by direct action or by stimulation of interleukin-1 secretion and is implicated in the induction of cachexia, Under certain conditions it can stimulate cell proliferation and induce cell differentiation. Impairs regulatory T-cells (Treg) function in individuals with rheumatoid arthritis via FOXP3 dephosphorylation. Upregulates the expression of protein phosphatase 1 [...]*	*Interleukin-6; Cytokine with a wide variety of biological functions. It is a potent inducer of the acute phase response. Plays an essential role in the final differentiation of B-cells into Ig-secreting cells Involved in lymphocyte and monocyte differentiation. Acts on B-cells, T-cells, hepatocytes, hematopoietic progenitor cells and cells of the CNS. Required for the generation of T(H)17 cells. Also acts as a myokine. It is discharged into the bloodstream after muscle contraction and acts to increase the breakdown of fats and to improve insulin resistance. It induces myeloma and plasm [...]*	0.994
**TNF**	PTGS2	ENSP00000398698	ENSP00000356438	*Tumor necrosis factor, membrane form; Cytokine that binds to TNFRSF1A/TNFR1 and TNFRSF1B/TNFBR. It is mainly secreted by macrophages and can induce cell death of certain tumor cell lines. It is potent pyrogen causing fever by direct action or by stimulation of interleukin-1 secretion and is implicated in the induction of cachexia, Under certain conditions it can stimulate cell proliferation and induce cell differentiation. Impairs regulatory T-cells (Treg) function in individuals with rheumatoid arthritis via FOXP3 dephosphorylation. Upregulates the expression of protein phosphatase 1 [...]*	*Prostaglandin G/H synthase 2; Converts arachidonate to prostaglandin H2 (PGH2), a committed step in prostanoid synthesis. Constitutively expressed in some tissues in physiological conditions, such as the endothelium, kidney and brain, and in pathological conditions, such as in cancer. PTGS2 is responsible for production of inflammatory prostaglandins. Up-regulation of PTGS2 is also associated with increased cell adhesion, phenotypic changes, resistance to apoptosis and tumor angiogenesis. In cancer cells, PTGS2 is a key step in the production of prostaglandin E2 (PGE2), which plays imp [...]*	0.916

Table S5: The interaction scores of the protein-protein interaction network for the investigated genes involved in apoptotic pathway.

node1	node2	node1 accession	node2 accession	node1 annotation	node2 annotation	score
**BAX**	BCL2	ENSP00000293288	ENSP00000381185	Apoptosis regulator BAX; Plays a role in the mitochondrial apoptotic process. Under normal conditions, BAX is largely cytosolic via constant retrotranslocation from mitochondria to the cytosol mediated by BCL2L1/Bcl-xL, which avoids accumulation of toxic BAX levels at the mitochondrial outer membrane (MOM). Under stress conditions, undergoes a conformation change that causes translocation to the mitochondrion membrane, leading to the release of cytochrome c that then triggers apoptosis. Promotes activation of CASP3, and thereby apoptosis.	Apoptosis regulator Bcl-2; Suppresses apoptosis in a variety of cell systems including factor-dependent lymphohematopoietic and neural cells. Regulates cell death by controlling the mitochondrial membrane permeability. Appears to function in a feedback loop system with caspases. Inhibits caspase activity either by preventing the release of cytochrome c from the mitochondria and/or by binding to the apoptosis-activating factor (APAF-1). May attenuate inflammation by impairing NLRP1-inflammasome activation, hence CASP1 activation and IL1B release.	0.999
**BAX**	CASP3	ENSP00000293288	ENSP00000311032	Apoptosis regulator BAX; Plays a role in the mitochondrial apoptotic process. Under normal conditions, BAX is largely cytosolic via constant retrotranslocation from mitochondria to the cytosol mediated by BCL2L1/Bcl-xL, which avoids accumulation of toxic BAX levels at the mitochondrial outer membrane (MOM). Under stress conditions, undergoes a conformation change that causes translocation to the mitochondrion membrane, leading to the release of cytochrome c that then triggers apoptosis. Promotes activation of CASP3, and thereby apoptosis.	Caspase-3 subunit p12; Involved in the activation cascade of caspases responsible for apoptosis execution. At the onset of apoptosis it proteolytically cleaves poly(ADP-ribose) polymerase (PARP) at a '216-Asp-|-Gly-217' bond. Cleaves and activates sterol regulatory element binding proteins (SREBPs) between the basic helix-loop-helix leucine zipper domain and the membrane attachment domain. Cleaves and activates caspase-6, -7 and -9. Involved in the cleavage of huntingtin. Triggers cell adhesion in sympathetic neurons through RET cleavage.	0.884
**BAX**	CYCS	ENSP00000293288	ENSP00000307786	Apoptosis regulator BAX; Plays a role in the mitochondrial apoptotic process. Under normal conditions, BAX is largely cytosolic via constant retrotranslocation from mitochondria to the cytosol mediated by BCL2L1/Bcl-xL, which avoids accumulation of toxic BAX levels at the mitochondrial outer membrane (MOM). Under stress conditions, undergoes a conformation change that causes translocation to the mitochondrion membrane, leading to the release of cytochrome c that then triggers apoptosis. Promotes activation of CASP3, and thereby apoptosis.	Cytochrome c; Electron carrier protein. The oxidized form of the cytochrome c heme group can accept an electron from the heme group of the cytochrome c1 subunit of cytochrome reductase. Cytochrome c then transfers this electron to the cytochrome oxidase complex, the final protein carrier in the mitochondrial electron-transport chain.	0.993
**BAX**	TP53	ENSP00000293288	ENSP00000269305	Apoptosis regulator BAX; Plays a role in the mitochondrial apoptotic process. Under normal conditions, BAX is largely cytosolic via constant retrotranslocation from mitochondria to the cytosol mediated by BCL2L1/Bcl-xL, which avoids accumulation of toxic BAX levels at the mitochondrial outer membrane (MOM). Under stress conditions, undergoes a conformation change that causes translocation to the mitochondrion membrane, leading to the release of cytochrome c that then triggers apoptosis. Promotes activation of CASP3, and thereby apoptosis.	Cellular tumor antigen p53; Acts as a tumor suppressor in many tumor types; induces growth arrest or apoptosis depending on the physiological circumstances and cell type. Involved in cell cycle regulation as a trans-activator that acts to negatively regulate cell division by controlling a set of genes required for this process. One of the activated genes is an inhibitor of cyclin-dependent kinases. Apoptosis induction seems to be mediated either by stimulation of BAX and FAS antigen expression, or by repression of Bcl-2 expression. Its pro-apoptotic activity is activated via its intera [...]	0.994
**BCL2**	BAX	ENSP00000381185	ENSP00000293288	Apoptosis regulator Bcl-2; Suppresses apoptosis in a variety of cell systems including factor-dependent lymphohematopoietic and neural cells. Regulates cell death by controlling the mitochondrial membrane permeability. Appears to function in a feedback loop system with caspases. Inhibits caspase activity either by preventing the release of cytochrome c from the mitochondria and/or by binding to the apoptosis-activating factor (APAF-1). May attenuate inflammation by impairing NLRP1-inflammasome activation, hence CASP1 activation and IL1B release.	Apoptosis regulator BAX; Plays a role in the mitochondrial apoptotic process. Under normal conditions, BAX is largely cytosolic via constant retrotranslocation from mitochondria to the cytosol mediated by BCL2L1/Bcl-xL, which avoids accumulation of toxic BAX levels at the mitochondrial outer membrane (MOM). Under stress conditions, undergoes a conformation change that causes translocation to the mitochondrion membrane, leading to the release of cytochrome c that then triggers apoptosis. Promotes activation of CASP3, and thereby apoptosis.	0.999

**BCL2**	CASP3	ENSP00000381185	ENSP00000311032	Apoptosis regulator Bcl-2; Suppresses apoptosis in a variety of cell systems including factor-dependent lymphohematopoietic and neural cells. Regulates cell death by controlling the mitochondrial membrane permeability. Appears to function in a feedback loop system with caspases. Inhibits caspase activity either by preventing the release of cytochrome c from the mitochondria and/or by binding to the apoptosis-activating factor (APAF-1). May attenuate inflammation by impairing NLRP1-inflammasome activation, hence CASP1 activation and IL1B release.	Caspase-3 subunit p12; Involved in the activation cascade of caspases responsible for apoptosis execution. At the onset of apoptosis it proteolytically cleaves poly(ADP-ribose) polymerase (PARP) at a '216-Asp-|-Gly-217' bond. Cleaves and activates sterol regulatory element binding proteins (SREBPs) between the basic helix-loop-helix leucine zipper domain and the membrane attachment domain. Cleaves and activates caspase-6, -7 and -9. Involved in the cleavage of huntingtin. Triggers cell adhesion in sympathetic neurons through RET cleavage.	0.987
**BCL2**	CYCS	ENSP00000381185	ENSP00000307786	Apoptosis regulator Bcl-2; Suppresses apoptosis in a variety of cell systems including factor-dependent lymphohematopoietic and neural cells. Regulates cell death by controlling the mitochondrial membrane permeability. Appears to function in a feedback loop system with caspases. Inhibits caspase activity either by preventing the release of cytochrome c from the mitochondria and/or by binding to the apoptosis-activating factor (APAF-1). May attenuate inflammation by impairing NLRP1-inflammasome activation, hence CASP1 activation and IL1B release.	Cytochrome c; Electron carrier protein. The oxidized form of the cytochrome c heme group can accept an electron from the heme group of the cytochrome c1 subunit of cytochrome reductase. Cytochrome c then transfers this electron to the cytochrome oxidase complex, the final protein carrier in the mitochondrial electron-transport chain.	0.997
**BCL2**	TP53	ENSP00000381185	ENSP00000269305	Apoptosis regulator Bcl-2; Suppresses apoptosis in a variety of cell systems including factor-dependent lymphohematopoietic and neural cells. Regulates cell death by controlling the mitochondrial membrane permeability. Appears to function in a feedback loop system with caspases. Inhibits caspase activity either by preventing the release of cytochrome c from the mitochondria and/or by binding to the apoptosis-activating factor (APAF-1). May attenuate inflammation by impairing NLRP1-inflammasome activation, hence CASP1 activation and IL1B release.	Cellular tumor antigen p53; Acts as a tumor suppressor in many tumor types; induces growth arrest or apoptosis depending on the physiological circumstances and cell type. Involved in cell cycle regulation as a trans-activator that acts to negatively regulate cell division by controlling a set of genes required for this process. One of the activated genes is an inhibitor of cyclin-dependent kinases. Apoptosis induction seems to be mediated either by stimulation of BAX and FAS antigen expression, or by repression of Bcl-2 expression. Its pro-apoptotic activity is activated via its intera [...]	0.999
**CASP3**	BAX	ENSP00000311032	ENSP00000293288	Caspase-3 subunit p12; Involved in the activation cascade of caspases responsible for apoptosis execution. At the onset of apoptosis it proteolytically cleaves poly(ADP-ribose) polymerase (PARP) at a '216-Asp-|-Gly-217' bond. Cleaves and activates sterol regulatory element binding proteins (SREBPs) between the basic helix-loop-helix leucine zipper domain and the membrane attachment domain. Cleaves and activates caspase-6, -7 and -9. Involved in the cleavage of huntingtin. Triggers cell adhesion in sympathetic neurons through RET cleavage.	Apoptosis regulator BAX; Plays a role in the mitochondrial apoptotic process. Under normal conditions, BAX is largely cytosolic via constant retrotranslocation from mitochondria to the cytosol mediated by BCL2L1/Bcl-xL, which avoids accumulation of toxic BAX levels at the mitochondrial outer membrane (MOM). Under stress conditions, undergoes a conformation change that causes translocation to the mitochondrion membrane, leading to the release of cytochrome c that then triggers apoptosis. Promotes activation of CASP3, and thereby apoptosis.	0.884
**CASP3**	BCL2	ENSP00000311032	ENSP00000381185	Caspase-3 subunit p12; Involved in the activation cascade of caspases responsible for apoptosis execution. At the onset of apoptosis it proteolytically cleaves poly(ADP-ribose) polymerase (PARP) at a '216-Asp-|-Gly-217' bond. Cleaves and activates sterol regulatory element binding proteins (SREBPs) between the basic helix-loop-helix leucine zipper domain and the membrane attachment domain. Cleaves and activates caspase-6, -7 and -9. Involved in the cleavage of huntingtin. Triggers cell adhesion in sympathetic neurons through RET cleavage.	Apoptosis regulator Bcl-2; Suppresses apoptosis in a variety of cell systems including factor-dependent lymphohematopoietic and neural cells. Regulates cell death by controlling the mitochondrial membrane permeability. Appears to function in a feedback loop system with caspases. Inhibits caspase activity either by preventing the release of cytochrome c from the mitochondria and/or by binding to the apoptosis-activating factor (APAF-1). May attenuate inflammation by impairing NLRP1-inflammasome activation, hence CASP1 activation and IL1B release.	0.987
**CASP3**	CYCS	ENSP00000311032	ENSP00000307786	Caspase-3 subunit p12; Involved in the activation cascade of caspases responsible for apoptosis execution. At the onset of apoptosis it proteolytically cleaves poly(ADP-ribose) polymerase (PARP) at a '216-Asp-|-Gly-217' bond. Cleaves and	Cytochrome c; Electron carrier protein. The oxidized form of the cytochrome c heme group can accept an electron from the heme group of the cytochrome c1 subunit of cytochrome reductase. Cytochrome c then transfers this electron to the cytochrome oxidase complex, the final protein	0.997

				activates sterol regulatory element binding proteins (SREBPs) between the basic helix-loop-helix leucine zipper domain and the membrane attachment domain. Cleaves and activates caspase-6, -7 and -9. Involved in the cleavage of huntingtin. Triggers cell adhesion in sympathetic neurons through RET cleavage.	carrier in the mitochondrial electron-transport chain.	
**CASP3**	TP53	ENSP00000311032	ENSP00000269305	Caspase-3 subunit p12; Involved in the activation cascade of caspases responsible for apoptosis execution. At the onset of apoptosis it proteolytically cleaves poly(ADP-ribose) polymerase (PARP) at a '216-Asp-|-Gly-217' bond. Cleaves and activates sterol regulatory element binding proteins (SREBPs) between the basic helix-loop-helix leucine zipper domain and the membrane attachment domain. Cleaves and activates caspase-6, -7 and -9. Involved in the cleavage of huntingtin. Triggers cell adhesion in sympathetic neurons through RET cleavage.	Cellular tumor antigen p53; Acts as a tumor suppressor in many tumor types; induces growth arrest or apoptosis depending on the physiological circumstances and cell type. Involved in cell cycle regulation as a trans-activator that acts to negatively regulate cell division by controlling a set of genes required for this process. One of the activated genes is an inhibitor of cyclin-dependent kinases. Apoptosis induction seems to be mediated either by stimulation of BAX and FAS antigen expression, or by repression of Bcl-2 expression. Its pro-apoptotic activity is activated via its intera [...]	0.956
**CYCS**	BAX	ENSP00000307786	ENSP00000293288	Cytochrome c; Electron carrier protein. The oxidized form of the cytochrome c heme group can accept an electron from the heme group of the cytochrome c1 subunit of cytochrome reductase. Cytochrome c then transfers this electron to the cytochrome oxidase complex, the final protein carrier in the mitochondrial electron-transport chain.	Apoptosis regulator BAX; Plays a role in the mitochondrial apoptotic process. Under normal conditions, BAX is largely cytosolic via constant retrotranslocation from mitochondria to the cytosol mediated by BCL2L1/Bcl-xL, which avoids accumulation of toxic BAX levels at the mitochondrial outer membrane (MOM). Under stress conditions, undergoes a conformation change that causes translocation to the mitochondrion membrane, leading to the release of cytochrome c that then triggers apoptosis. Promotes activation of CASP3, and thereby apoptosis.	0.993
**CYCS**	BCL2	ENSP00000307786	ENSP00000381185	Cytochrome c; Electron carrier protein. The oxidized form of the cytochrome c heme group can accept an electron from the heme group of the cytochrome c1 subunit of cytochrome reductase. Cytochrome c then transfers this electron to the cytochrome oxidase complex, the final protein carrier in the mitochondrial electron-transport chain.	Apoptosis regulator Bcl-2; Suppresses apoptosis in a variety of cell systems including factor-dependent lymphohematopoietic and neural cells. Regulates cell death by controlling the mitochondrial membrane permeability. Appears to function in a feedback loop system with caspases. Inhibits caspase activity either by preventing the release of cytochrome c from the mitochondria and/or by binding to the apoptosis-activating factor (APAF-1). May attenuate inflammation by impairing NLRP1-inflammasome activation, hence CASP1 activation and IL1B release.	0.997
**CYCS**	CASP3	ENSP00000307786	ENSP00000311032	Cytochrome c; Electron carrier protein. The oxidized form of the cytochrome c heme group can accept an electron from the heme group of the cytochrome c1 subunit of cytochrome reductase. Cytochrome c then transfers this electron to the cytochrome oxidase complex, the final protein carrier in the mitochondrial electron-transport chain.	Caspase-3 subunit p12; Involved in the activation cascade of caspases responsible for apoptosis execution. At the onset of apoptosis it proteolytically cleaves poly(ADP-ribose) polymerase (PARP) at a '216-Asp-|-Gly-217' bond. Cleaves and activates sterol regulatory element binding proteins (SREBPs) between the basic helix-loop-helix leucine zipper domain and the membrane attachment domain. Cleaves and activates caspase-6, -7 and -9. Involved in the cleavage of huntingtin. Triggers cell adhesion in sympathetic neurons through RET cleavage.	0.997
**CYCS**	TP53	ENSP00000307786	ENSP00000269305	Cytochrome c; Electron carrier protein. The oxidized form of the cytochrome c heme group can accept an electron from the heme group of the cytochrome c1 subunit of cytochrome reductase. Cytochrome c then transfers this electron to the cytochrome oxidase complex, the final protein carrier in the mitochondrial electron-transport chain.	Cellular tumor antigen p53; Acts as a tumor suppressor in many tumor types; induces growth arrest or apoptosis depending on the physiological circumstances and cell type. Involved in cell cycle regulation as a trans-activator that acts to negatively regulate cell division by controlling a set of genes required for this process. One of the activated genes is an inhibitor of cyclin-dependent kinases. Apoptosis induction seems to be mediated either by stimulation of BAX and FAS antigen expression, or by repression of Bcl-2 expression. Its pro-apoptotic activity is activated via its intera [...]	0.911
**TP53**	BAX	ENSP00000269305	ENSP00000293288	Cellular tumor antigen p53; Acts as a tumor suppressor in many tumor types; induces growth arrest or apoptosis depending on the physiological circumstances and cell type. Involved in cell cycle regulation as a trans-activator that acts to negatively regulate cell division by controlling a set of genes required for this process. One of the activated genes is an inhibitor of cyclin-	Apoptosis regulator BAX; Plays a role in the mitochondrial apoptotic process. Under normal conditions, BAX is largely cytosolic via constant retrotranslocation from mitochondria to the cytosol mediated by BCL2L1/Bcl-xL, which avoids accumulation of toxic BAX levels at the mitochondrial outer membrane (MOM). Under stress conditions, undergoes a conformation change that causes translocation to the mitochondrion membrane, leading to	0.994

				dependent kinases. Apoptosis induction seems to be mediated either by stimulation of BAX and FAS antigen expression, or by repression of Bcl-2 expression. Its pro-apoptotic activity is activated via its intera [...]	the release of cytochrome c that then triggers apoptosis. Promotes activation of CASP3, and thereby apoptosis.	
**TP53**	BCL2	ENSP00000269305	ENSP00000381185	Cellular tumor antigen p53; Acts as a tumor suppressor in many tumor types; induces growth arrest or apoptosis depending on the physiological circumstances and cell type. Involved in cell cycle regulation as a trans-activator that acts to negatively regulate cell division by controlling a set of genes required for this process. One of the activated genes is an inhibitor of cyclin-dependent kinases. Apoptosis induction seems to be mediated either by stimulation of BAX and FAS antigen expression, or by repression of Bcl-2 expression. Its pro-apoptotic activity is activated via its intera [...]	Apoptosis regulator Bcl-2; Suppresses apoptosis in a variety of cell systems including factor-dependent lymphohematopoietic and neural cells. Regulates cell death by controlling the mitochondrial membrane permeability. Appears to function in a feedback loop system with caspases. Inhibits caspase activity either by preventing the release of cytochrome c from the mitochondria and/or by binding to the apoptosis-activating factor (APAF-1). May attenuate inflammation by impairing NLRP1-inflammasome activation, hence CASP1 activation and IL1B release.	0.999
**TP53**	CASP3	ENSP00000269305	ENSP00000311032	Cellular tumor antigen p53; Acts as a tumor suppressor in many tumor types; induces growth arrest or apoptosis depending on the physiological circumstances and cell type. Involved in cell cycle regulation as a trans-activator that acts to negatively regulate cell division by controlling a set of genes required for this process. One of the activated genes is an inhibitor of cyclin-dependent kinases. Apoptosis induction seems to be mediated either by stimulation of BAX and FAS antigen expression, or by repression of Bcl-2 expression. Its pro-apoptotic activity is activated via its intera [...]	Caspase-3 subunit p12; Involved in the activation cascade of caspases responsible for apoptosis execution. At the onset of apoptosis it proteolytically cleaves poly(ADP-ribose) polymerase (PARP) at a '216-Asp-|-Gly-217' bond. Cleaves and activates sterol regulatory element binding proteins (SREBPs) between the basic helix-loop-helix leucine zipper domain and the membrane attachment domain. Cleaves and activates caspase-6, -7 and -9. Involved in the cleavage of huntingtin. Triggers cell adhesion in sympathetic neurons through RET cleavage.	0.956
**TP53**	CYCS	ENSP00000269305	ENSP00000307786	Cellular tumor antigen p53; Acts as a tumor suppressor in many tumor types; induces growth arrest or apoptosis depending on the physiological circumstances and cell type. Involved in cell cycle regulation as a trans-activator that acts to negatively regulate cell division by controlling a set of genes required for this process. One of the activated genes is an inhibitor of cyclin-dependent kinases. Apoptosis induction seems to be mediated either by stimulation of BAX and FAS antigen expression, or by repression of Bcl-2 expression. Its pro-apoptotic activity is activated via its intera [...]	Cytochrome c; Electron carrier protein. The oxidized form of the cytochrome c heme group can accept an electron from the heme group of the cytochrome c1 subunit of cytochrome reductase. Cytochrome c then transfers this electron to the cytochrome oxidase complex, the final protein carrier in the mitochondrial electron-transport chain.	0.911

References

1. Kanehisa M, Goto S. KEGG: kyoto encyclopedia of genes and genomes. Nucleic Acids Res. 2000;28(1):27-30.
2. Hendrata M, Sudiono J. A Computational Model for Investigating Tumor Apoptosis Induced by Mesenchymal Stem Cell-Derived Secretome. Computational and mathematical methods in medicine. 2016;2016:4910603.
3. Szklarczyk D, Gable AL, Nastou KC, Lyon D, Kirsch R, Pyysalo S, et al. The STRING database in 2021: customizable protein-protein networks, and functional characterization of user-uploaded gene/measurement sets. Nucleic Acids Res. 2021;49(D1):D605-d12.
4. Arulselvan P, Tan WS, Gothai S, Muniandy K, Fakurazi S, Esa NM, et al. Anti-Inflammatory Potential of Ethyl Acetate Fraction of Moringa oleifera in Downregulating the NF-κB Signaling Pathway in Lipopolysaccharide-Stimulated Macrophages. Molecules (Basel, Switzerland). 2016;21(11).
5. Khan MM, Gandhi C, Chauhan N, Stevens JW, Motto DG, Lentz SR, et al. Alternatively-spliced extra domain A of fibronectin promotes acute inflammation and brain injury after cerebral ischemia in mice. Stroke. 2012;43(5):1376-82.
